# Predator movements in relation to habitat features reveal vulnerability of duck nests to predation

**DOI:** 10.1002/ece3.9329

**Published:** 2022-09-20

**Authors:** Sarah H. Peterson, Joshua T. Ackerman, Meghan P. Keating, Carley R. Schacter, C. Alex Hartman, Michael L. Casazza, Mark P. Herzog

**Affiliations:** ^1^ U.S. Geological Survey, Western Ecological Research Center, Dixon Field Station Dixon California USA

**Keywords:** dabbling duck, marsh, mesopredator, nest survival, predator–prey interaction

## Abstract

Nest predation is the main cause of nest failure for ducks. Understanding how habitat features influence predator movements may facilitate management of upland and wetland breeding habitats that reduces predator encounter rates with duck nests and increases nest survival rates. For 1618 duck nests, nest survival increased with distance to phragmites (*Phragmites australis*), shrubs, telephone poles, human structures, and canals, but not for four other habitat features. Using GPS collars, we tracked 25 raccoons (*Procyon lotor*) and 16 striped skunks (*Mephitis mephitis*) over 4 years during waterfowl breeding and found marked differences in how these predators were located relative to specific habitat features; moreover, the probability of duck nests being encountered by predators differed by species. Specifically, proximity to canals, wetlands, trees, levees/roads, human structures, shrubs, and telephone poles increased the likelihood of a nest being encountered by collared raccoons. For collared skunks, nests were more likely to be encountered if they were closer to canals, trees, and shrubs, and farther from wetlands and human structures. Most predator encounters with duck nests were attributable to a few individuals; 29.2% of raccoons and 38.5% of skunks were responsible for 95.6% of total nest encounters. During the central span of duck nesting (April 17–June 14: 58 nights), these seven raccoons and five skunks encountered >1 nest on 50.8 ± 29.2% (mean ± SD) and 41.5 ± 28.3% of nights, respectively, and of those nights individual raccoons and skunks averaged 2.60 ± 1.28 and 2.50 ± 1.09 nest encounters/night, respectively. For collared predators that encountered >1 nest, a higher proportion of nests encountered by skunks had evidence of predation (51.9 ± 26.6%) compared to nests encountered by raccoons (22.3 ± 17.1%). Because duck eggs were most likely consumed as raccoons and skunks opportunistically discovered nests, managing the habitat features those predators most strongly associated with could potentially reduce rates of egg predation.

## Graphical abstract

Nest predation is the primary cause of nest failure for ducks; thus, quantifying predator‐specific movement behavior can clarify the vulnerability of individual nests to predation in heterogenous landscapes. Using GPS collars, we simultaneously tracked mesopredators at a fine spatial and temporal scale and monitored duck nest availability and nest fate on the landscape. We designed a series of questions to test how raccoons and striped skunks associated with habitat features in upland and wetland habitats and the implications of the foraging movements for nesting waterfowl.

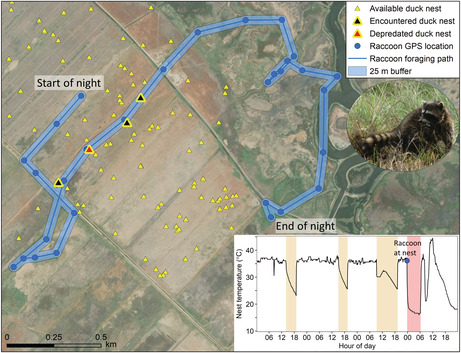

## 1 INTRODUCTION

Landscape heterogeneity and habitat characteristics can have substantial effects on predator movements and the vulnerability of target and non‐target prey species (Gorini et al., 2012). In heterogenous environments, both predator and prey species can associate differently with habitat features. For example, habitat features such as roads or trails may serve as travel corridors for predators and be avoided by prey species (DeGregorio et al., 2014; Dickie et al., 2020; James & Stuart‐Smith, 2000). Some predator species demonstrate a preference for foraging along habitat edges, and bird nests in some habitats near to edges with focused predator movements can experience greater predation risk (Hannon & Cotterill, 1998; Ibarzabal & Desrochers, 2004).

At the landscape level, the effect of distance to habitat edges on avian nest survival has been largely equivocal, with many studies not detecting effects and other studies finding effects in some, but not all, treatments (reviewed in Lahti, 2001; Vetter et al., 2013). Importantly, when real differences in predation risk exist in an ecosystem as a function of how nests are distributed in relation to specific habitat features, the failure to detect effects may in part be attributed to the scale of study and differences in species‐specific predator behaviors that in essence act in opposition to each other. Studies that examined predation rates at the landscape scale tended not to find an influence of distance to edge habitat effects on nest survival, whereas studies examining predation rates at smaller spatial scales that also accounted for species‐specific predator behaviors detected effects more often (reviewed in Lahti, 2001). Therefore, studies that combine specific, more refined predator movements on the landscape with nest survival of birds may be more likely to detect effects of proximity to certain habitats, including edge habitats and possible predator corridors, on the vulnerability of bird nests to predation.

Dabbling ducks often nest at relatively high densities in upland habitats (McLandress et al., 1996) and need access to nearby wetlands during egg incubation for their daily nest breaks (Croston et al., 2020) and during brood rearing (Casazza et al., 2020; Mauser et al., 1994). For waterfowl nesting within highly managed upland nesting areas, the requirement for both upland and wetland habitats to be in relatively close proximity during breeding often results in a heterogenous landscape with many edge habitats, including features such as roads, levees, canals, and wetland edges. Predation of eggs by mammalian and avian predators is the main cause of waterfowl nest failure and high levels of nest predation can limit population growth (Cowardin et al., 1985; Hoekman et al., 2002; Klett et al., 1988; Sargeant & Raveling, 1992). If predators are typically located closer to certain habitat features than others (Barding & Nelson, 2008; Bixler & Gittleman, 2000; DeGregorio et al., 2014; Fritzell, 1978; Greenwood, 1982; Roos, 2002; Storm, 1972), the fine‐scale location of prey, such as duck eggs, in relation to certain habitat features, may influence the vulnerability of individual nests to egg predation. For example, if a predator moves and forages primarily along wetland edges, then duck nests in upland areas that are closer to wetlands may have a higher probability of being encountered opportunistically, even if predators are not searching for duck nests specifically.

The likelihood of predators encountering and consuming prey resources is influenced by a combination of predator movement patterns, habitat structure, the location of prey resources on the landscape, predator search behavior, and prey characteristics such as camouflage or other predator avoidance behaviors. Seasonally available prey that are available for only a few months out of the year, such as dabbling duck eggs, may elicit behavioral responses by predators. Predators may alter their foraging efforts to search for more of an ephemeral prey resource (functional response) or aggregate in locations where densities of the prey resource are high (aggregative response). The magnitude of a predator response to an ephemeral prey resource (e.g., nests) can be influenced by the density of the resource (Ackerman et al., 2004; Holling, 1959; Larivière & Messier, 1998; Ringelman et al., 2014; Roos, 2002; Schmidt & Whelan, 1998) as well as the availability of alternate prey (Ackerman, 2002; Crabtree & Wolfe, 1988; Korpimäki et al., 1991; McKinnon et al., 2014). Predators also might not markedly alter their movements or general prey searching behaviors in response to a seasonal shift in prey resources, and instead consume seasonally available prey only opportunistically when encountered (Husby & Hoset, 2018; Urban, 1970), particularly if the prey resource is relatively difficult to locate.

Given that predator species may associate with different key habitat features, such as wetland edges or roads, examining predator‐specific movement behavior at a fine spatial and temporal scale may clarify the vulnerability of individual nests to predation. We designed a series of questions related to fine‐scale predator movements and the vulnerability of dabbling duck nests using two of the most ubiquitous predators of waterfowl eggs in North America, raccoons (*Procyon lotor*) and striped skunks (*Mephitis mephitis*; Croston, Ackerman, et al., 2018; Klett et al., 1988; Sargeant et al., 1998; Sargeant et al., 1995; Sargeant & Raveling, 1992). First, we compared the locations of duck nests and predator movement locations (GPS collars on raccoons and striped skunks) relative to key habitat features within a core block of upland nesting habitat to better describe the proximity of individual predators and nests to specific habitat features. Next, we tested if nest survival increased with distance to certain habitat features on the landscape, including those that were used to a greater extent by predators. We then linked the individual nightly movements of tracked raccoons and striped skunks with nest encounters and the fate of duck nests. Finally, we used nests within the known home ranges of collared predators to examine species‐specific relationships between the vulnerability of individual duck nests to encounters with tracked predators based on the proximity to key habitat features.

## 2 METHODS

### 2.1 Field sampling and data collection

#### 2.1.1 Study site

**FIGURE 1:**
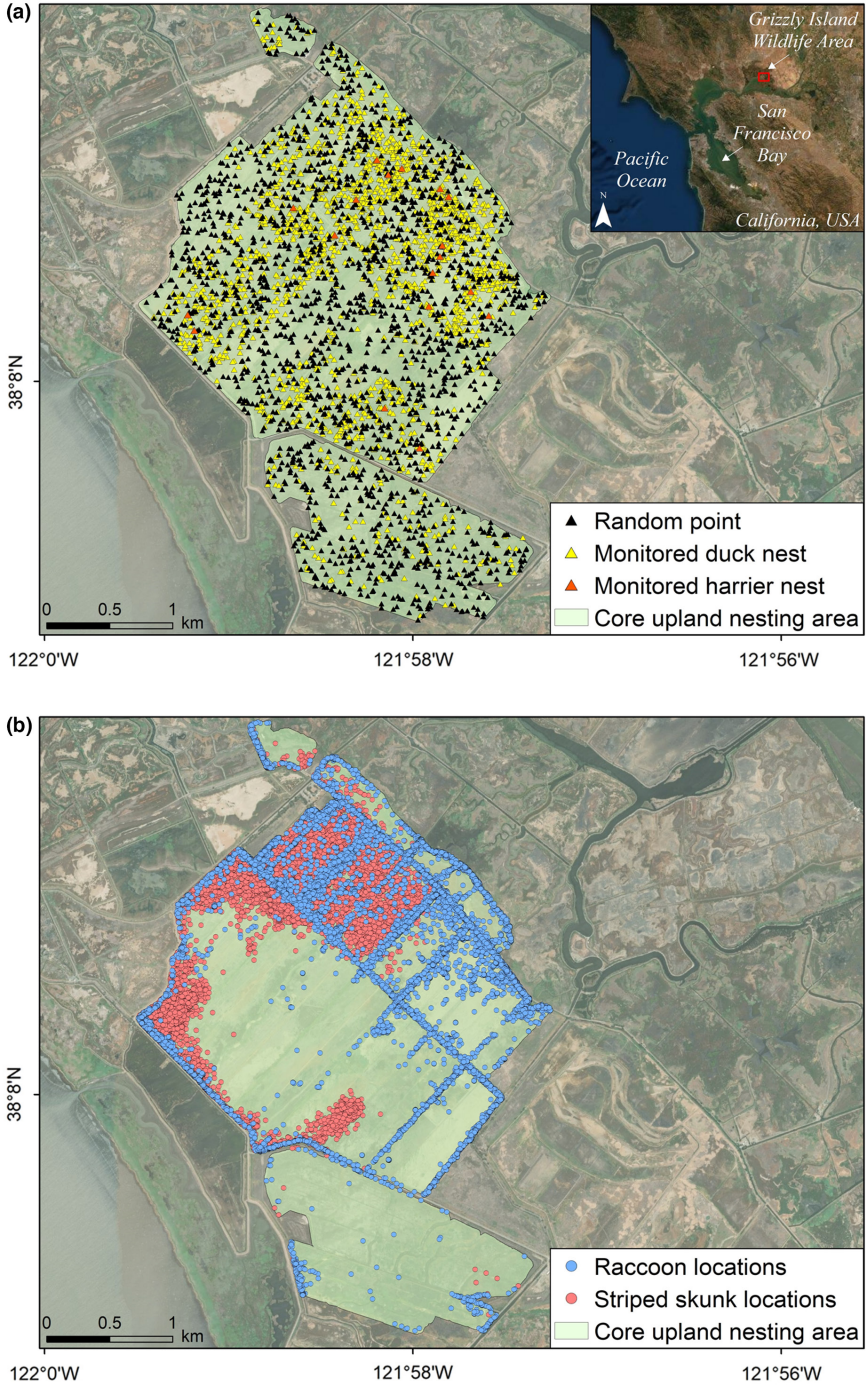
(a) Monitored duck nests (yellow triangles), northern harrier nests (*Circus hudsonius*; orange triangles) and random locations (black triangles) within the core upland nesting area of the publicly managed Grizzly Island Wildlife Area and on adjacent privately owned land (Suisun Marsh, California, 2016–2019). (b) GPS locations of collared raccoons (*Procyon lotor*; blue circles) and striped skunks (*Mephitis mephitis*, red circles) when animals were located within the core upland duck nesting area.

We conducted this study from 2016 to 2019 in Suisun Marsh, a large and extensively managed brackish marsh located in the Sacramento‐San Joaquin Delta of California (USA). Upland habitats within Suisun Marsh, mostly within a publicly managed wildlife area (Grizzly Island Wildlife Area; 38.141°N, 121.970°W; Figure 1), provide important waterfowl and northern harrier (*Circus hudsonius*) nesting habitat (Ackerman et al., 2014; McLandress et al., 1996). Northern harriers are a species of special concern in California (Shuford & Gardali, 2008). We monitored nests within a 7.1 km^2^ block of primarily upland habitat managed by the California Department of Fish and Wildlife and adjacent private landowners (hereafter referred to as the core upland nesting area). Upland vegetation within the core upland nesting area includes a range of species such as mid‐height (<1 m) grasses (*Lolium* spp., *Hordeum* spp., *Bromus* spp., *Polypogon monspeliensis*), taller (>1 m) grasses (*Elytrigia* spp., *Phalaris* spp.), vetch (*Vicia* spp), herbs (*Atriplex patula*, *Lotus corniculatus*), thistle (family Asteraceae), and pickleweed (*Salicornia virginica*). Individually managed upland fields within the core upland nesting area are separated by roads, drivable levees, elevated dirt levees, and water transportation ditches (Ackerman et al., 2004; Raquel et al., 2015). Predators were captured on and immediately adjacent to the Grizzly Island Wildlife Area.

#### 2.1.2 Nest searching and monitoring

We used standard nest‐searching protocols, modified from McLandress et al. (1996) to find dabbling duck nests in upland habitats from March to July 2016 to 2019; every 3 weeks, we searched upland habitat with a rope and attached cans pulled between two all‐terrain vehicles (ATVs). For each nest, we identified the species visually when a hen flushed off the nest as well as by the size and color of the eggs. We marked and monitored nests of all ground‐nesting non‐passerine species and revisited nests weekly to monitor nest development (by candling eggs; Weller, 1956), determine nest fate (e.g., hatched, depredated), and document any evidence of predation (e.g., eggshells or missing eggs). We estimated the nest initiation date by subtracting the average incubation stage and clutch size at discovery from the date of discovery. In 2016 and 2017, nests were visited weekly until the nest hatched or failed and then nest visits ceased; in 2018 and 2019, all nests were monitored weekly until after the nesting season (July 20th or 29th, respectively) if any eggs remained in the nest and regardless of whether the hen was still tending to the nest. After each nest monitoring visit, eggs were covered with down feathers and other nesting material at active nests (hen still tending to the nest), to mimic what hens typically do when they leave for an incubation recess, and eggs were left as they were found (covered or uncovered) after nests were confirmed to be inactive. With a nest‐searching interval of 3 weeks, as described above, and the high probability that many nests were initiated and failed within these 21 days, counts of discovered nests are minimum counts of nests in the study area (Johnson, 1979).

#### 2.1.3 Nest temperature loggers

In order to determine if a hen was present and flushed from the nest when a collared predator approached the nest, we used small data loggers placed within each duck nest to collect nest temperature data (Croston et al., 2021; Croston, Hartman, et al., 2018). When a nest was first found, we deployed an iButton temperature datalogger (Model DS1922L‐F5#, Maxim Integrated Products, Inc.) in the center of the nest bowl, flush with the apical surface of the eggs. To record ambient temperature at each nest, a second datalogger was deployed just south of the nest bowl. All iButtons were preprogrammed to collect data at 8‐min intervals. We used monotonic decreases in the nest temperature to identify when the hen left the nest (incubation recess; Croston, Hartman, et al., 2018). Previously, Croston et al. (2021) found that mallard (*Anas platyrhynchos*) and gadwall (*Mareca strepera*) nest temperatures decreased faster when hens did not cover their eggs prior to departure from a nest at night. Hence, we used this rate of temperature decrease to predict if nest departures during dusk and night hours were hen initiated (eggs were covered and had a low rate of temperature decrease) or predator initiated (hen flushed from the nest, eggs were left uncovered and had a high rate of temperature decrease) (Croston et al., 2021). Additionally, nest temperature data were used to determine the date and time the hen left the nest for the final time.

#### 2.1.4 Raccoon and skunk capture and collar deployments

To quantify animal movements in relation to nests and habitat features during the duck‐nesting period from mid‐March through July, we deployed two types of combined global positioning system (GPS) and very high frequency (VHF) collars (Table 1). We were generally able to capture individual raccoons only once; thus, we deployed a collar (W500 Wildlink GPS Logger; Advanced Telemetry Systems) that allowed for remote downloading of the data. The raccoon collar was powered using a C‐sized battery and weighed approximately 120–138 g. In 2016 only, raccoon collars were powered using a single AA battery and weighed approximately 65 g. Skunks were frequently recaptured; therefore, we used an archival collar that stored data on board (G10 UltraLITE collar; Advanced Telemetry Systems) coupled with a VHF transmitter (Advanced Telemetry Systems). Skunk collars weighed approximately 26–30 g. We captured raccoons and skunks mostly in winter (January–March) and recaptured skunks mostly in summer (June and July) from 2016 to 2019 (Table 1). Skunk GPS deployments did not begin until 2018, due to technical difficulties with the initial collar design and manufacturer. Animal trapping and chemical immobilization procedures were described previously (Peterson et al., 2021).

**TABLE 1:** Raccoon (*Procyon lotor*) and striped skunk (*Mephitis mephitis*) equipped with GPS collars during the waterfowl nesting season in Suisun Marsh, California (Grizzly Island Wildlife Area) during 2016–2019.

Sex	Year	Animal ID	Mass at capture (kg)	Collar deploy date	Last date of GPS locations	Foraging nights	GPS locations	Percent of locations in core upland area	Nests in home range	Nests in home range while tracked	Encountered nests (25 m)	Encountered nests with predation (25 m)	Percent nests with predation
Raccoon													
Female	2016	746^a^	4.96	4/11/2016	5/31/2016	50	2427	37.9	60	43	5	0	0
Female	2017	48	6.58	2/13/2017	8/1/2017	135	4453	5.2	4	4	0	—	—
Female	2017	55	7.50	2/15/2017	8/1/2017	137	4147	17.7	35	35	1	1	100.0
Female	2017	65	5.38	2/24/2017	8/1/2017	139	5148	34.5	177	177	33	8	24.2
Female	2018	114	4.10	2/9/2018	8/1/2018	119	4814	35.6	145	145	12	2	16.7
Female	2018	122	4.90	2/8/2018	5/11/2018	56	2166	0.1	1	0	—	—	—
Female	2019	309	6.90	7/23/2018^c^	5/29/2019	69	2107	29.3	32	23	1	0	0
Female	2019	319	5.00	2/20/2019	7/26/2019	128	4982	52.1	133	133	41	17	41.5
Female	2019	321	6.10	2/6/2019	8/1/2019	134	5198	2.2	11	11	0	—	—
Female	2019	325	5.22	2/6/2019	8/1/2019	137	3829	0.8	2	2	0	—	—
Female	2019	327	6.60	2/11/2019	6/12/2019	89	3551	3.0	4	4	0	—	—
Female	2019	329	5.80	2/12/2019	8/1/2019	136	4212	1.7	2	2	0	—	—
Female	2019	333	5.40	2/7/2019	8/1/2019	131	3302	<0.1	2	2	0	—	—
Female	2019	335	6.20	2/12/2019	8/1/2019	134	4350	1.5	3	3	0	—	—
Male	2016	50^a^	6.94	4/28/2016	8/1/2016	95	4800	30.8	250	204	38	19	50.0
Male	2017	42	7.74	2/11/2017	5/7/2017	49	2220	2.3	5	5	0	—	—
Male	2017	63	7.22	2/24/2017	8/1/2017	139	5309	57.0	294	294	145	54	37.2
Male	2017	67	6.70	2/24/2017	8/1/2017	133	4567	2.2	4	4	0	—	—
Male	2018	103	6.90	2/8/2018	8/1/2018	139	5677	6.0	37	37	4	1	25.0
Male	2018	124	5.18	2/13/2018	8/1/2018	126	5270	7.6	9	9	2	0	0
Male	2018	128	6.74	2/16/2018	5/11/2018	57	2693	0.2	1	0	—	—	—
Male	2018	130	7.82	2/17/2018	7/18/2018	92	3883	13.5	48	48	14	1	7.1
Male	2019	197	7.96	7/13/2018^c^	4/9/2019	25	1118	7.7	48	3	0	—	—
Male	2019	304^b^	6.68	7/17/2018^c^	3/30/2019	15	675	9.9	35	0	—	—	—
Male	2019	317	6.26	2/6/2019	5/30/2019	76	3215	28.5	229	196	48	10	20.8
Striped skunk													
Female	2018	116	1.16	3/7/2018	5/8/2018	53	1249	100.0	59	30	6	5	83.3
Female	2018	149	1.20	1/30/2018	5/31/2018	72	2096	72.5	15	12	3	3	100.0
Female	2018	69	1.34	1/30/2018	6/30/2018	91	387	93.5	83	83	13	6	46.2
Female	2018	95^b^	1.58	2/28/2018	4/6/2018	22	1145	75.8	47	6	0	—	—
Female	2019	306	1.12	2/21/2019	6/5/2019	80	2787	79.4	91	84	34	17	50.0
Female	2019	339	1.20	2/21/2019	4/20/2019	31	1363	79.2	67	6	1	0	0
Female	2019	349	1.32	3/5/2019	7/16/2019	82	1422	67.9	173	173	65	26	40.0
Female	2019	351	1.36	3/9/2019	6/12/2019	87	3248	45.0	57	54	12	3	25.0
Male	2018	108	1.80	1/30/2018	4/18/2018	34	1336	6.0	6	0	—	—	—
Male	2018	39	2.30	1/26/2018	5/9/2018	51	1066	26.7	26	14	4	1	25.0
Male	2018	59	1.56	2/11/2018	5/15/2018	61	2453	23.9	15	8	0	—	—
Male	2019	106	1.80	3/4/2019	6/23/2019	99	1782	54.7	156	155	44	20	45.5
Male	2019	120^b^	1.48	2/25/2019	4/1/2019	17	632	82.4	47	2	0	—	—
Male	2019	323	—	2/6/2019	6/24/2019	101	5689	0.0	0	0	—	—	—
Male	2019	341	1.80	2/24/2019	5/10/2019	56	2700	0.3	6	3	0	—	—
Male	2019	92^b^	1.44	3/7/2019	4/2/2019	18	703	71.3	70	2	0	—	—

*Note*: GPS locations and the number of foraging nights used in this study started on March 15 except for two raccoons collared in 2016.

^a^ The percent of encountered nests with evidence of predation at the subsequent nest monitoring visit (percent nests with predation) was only calculated if at least one nest was encountered.

^b^ Several individuals were excluded from fine‐scale interactions with duck nests, as they did not have more than a week of locations after the start of duck nest monitoring.

^c^ Three raccoons were captured in the summer of 2018 and equipped with GPS collars that were programmed to sample infrequently until the subsequent duck nesting season in 2019.

#### 2.1.5 Raccoon GPS location acquisition and processing

To conserve battery life and maximize GPS data acquisition around duck nesting, we preprogrammed raccoon collars to initially collect two daily locations (midnight and noon) for several weeks after deployment. After this, location acquisition increased to collect locations daily every 15 min for 15 h (1800–0900 h Pacific Standard Time; GMT‐8 h), when most raccoon and skunk movements and predation of duck nests occur (Croston, Ackerman, et al., 2018), in addition to collecting two midday locations. We used VHF signals to locate raccoons and ultra‐high frequency (UHF) technology to remotely download data approximately weekly. To fill in missing GPS locations at night, we interpolated using the moveHMM R package (Michelot et al., 2016). Any night with >50% missing locations was visually inspected, as well as the preceding and subsequent nights, and we removed the entire night of foraging if tag failure, rather than animal behavior (i.e., denning behavior), was the probable cause behind reduced acquisition of locations. We also excluded the rare sequences of interpolated locations that occurred at the start or end of a night because an animal moved during the day. To align with duck nesting, locations were excluded prior to March 15 and after July 31. Raccoon locations were acquired daily from March 15 to the night of July 31 for 53.8% (*n =* 14) of collar deployments (Table 1). GPS data were fully censored from six raccoons for 1, 4, 13, 20, 33, and 45 days each.

To identify locations associated with nightly movements and avoid including locations that were associated with the daytime resting site (Fritzell, 1978), we used a distance threshold of 20 m between consecutive locations in a 15 min period (step length) at the start and end of the night. We evaluated different possible step lengths and decided that 20 m best captured actual departures from the day resting site while minimizing false positives. First, we removed locations at the start of the night until we reached the first step length between locations >20 m. Next, we did the same process at the end of the night moving backward from the last location of the night, removing locations until we reached a step length >20 m. This reduced dataset of locations gave us all nightly locations when a raccoon was moving between day resting sites, although animals were not necessarily moving over that entire period. We used this subset of locations once raccoons began moving for the night to examine potential encounters with all monitored bird nests and quantify the boundaries of the area covered by individual animals (range: 675–5677 locations per raccoon, due to differences in tracking duration; Table 1; determination of individual boundaries is described later in the methods). Additionally, we estimated the minimum distance traveled by each animal each night between day resting sites using the sum of the step lengths for that night.

#### 2.1.6 Skunk GPS location acquisition and processing

We collected and processed skunk data differently than for raccoons due to differences in the tag capabilities and acquisition of locations. Skunk collars were preprogrammed to turn on every 7.5 min for 512 ms 24 h/day to snap an image of the sky position of GPS satellites and then snaps were post‐processed and solved to GPS locations once the collars were recovered. After visually inspecting the data to determine appropriate thresholds, we used a filter for speed (5500 m/h; >99.7% of speeds) and step length (1600 m between consecutive locations) to systematically remove erroneous GPS locations (0.2% of locations), as the method of obtaining GPS locations on the skunk collars results in lower positional accuracy than a standard GPS collar (Elfelt & Moen, 2014). We then visually inspected tracks and censored biologically unlikely locations that passed through the speed and step length filter but were improbable based on preceding and subsequent locations (*n =* 6 locations). Locations for skunks were excluded prior to March 15 and the last skunk collar was recovered on July 16 (Table 1). Skunks routinely enter and exit burrows with narrow entrances, which leads to antenna breakage and reduces the quality of GPS and VHF data. We added a neoprene sleeve around the antennas in 2019 that helped to prolong antenna functionality, although by the time of recapture (2–5 months after initial tagging), every antenna was broken off the units to some degree, which decreased the number of locations we obtained, particularly because skunks tend to spend time in and under dense vegetation. Thus, we examined each individual animal separately and censored locations after the point in time when there was a substantial decrease in the number of GPS locations per day, indicating the day when the GPS antenna likely broke. Finally, we removed daytime locations between 0900 and 1800 h (GMT‐8 h) to have a comparable dataset to raccoons and exclude the period of the day when mammalian interactions with duck nests were unlikely (Croston, Ackerman, et al., 2018). We did not have the data resolution to exclude locations associated with day resting sites for skunks. The final dataset included a range of 387–5689 locations per skunk. As with raccoons, we quantified the minimum nightly distance traveled by each animal as the sum of the step lengths each night.

#### 2.1.7 Digitizing habitat features

We first identified a set of specific habitat features, including some edge habitats such as levees/roads, canals, and wetland edges, that we hypothesized could influence the likelihood of duck nest predation by influencing predator movements. We digitized all habitat features from the following categories: (1) levee/road (all berms between management units of upland habitat as well as drivable levees and roads), (2) ATV path (narrow path through upland habitat created in the process of searching for duck nests), (3) tree, (4) phragmites (*Phragmites australis*) patch, (5) shrub patch (*Atriplex lentiformis* and *Baccharis pilularis*), (6) telephone pole, and (7) other human structure (e.g., house, shed, large dumpster). Additionally, we digitized (8) the edge (water boundary) of seasonal wetlands and (9) water transportation features (such as shallow water transport ditches that ran between units of upland habitat; hereafter ‘canals’). For some analyses we also created a 10th habitat category, drivable levee/road, that was a subset of levees/roads where the levee/road was wide, maintained frequently, and passable for a four‐wheel drive truck. Habitat features were delineated using a combination of satellite imagery (Planet Labs™, Google Earth™, Digital Globe™) and a 2015 Suisun Marsh vegetation layer (California Department of Fish and Wildlife: https://gis.data.ca.gov/datasets/CDFW::vegetation‐suisun‐marsh‐2015‐ds2676‐1/about). Phragmites and shrub patches were typically large enough to be visible in satellite imagery (>5 m in diameter), but we also used real‐time kinematic GPS (Leica Smart Rover GPS1200, Leica Geosystems) to ground truth the edge of these smaller habitat features that may have less visible using satellite imagery. The digitizing of water features is described in detail within Schacter et al. (2021). Briefly, for seasonal wetlands we used satellite‐derived imagery to digitize the extent of water at three times during the waterfowl nesting period for each year: April (early nesting), May (mid nesting), and July (late nesting). For canals, we modified a layer of canals initially created by the Bay Area Aquatic Resources Inventory (San Francisco Estuary Institute and Aquatic Science Center (SFEI ASC), 2017) by actively ground‐truthing the imagery layer during duck nesting.

### 2.2 Experimental design and research questions

#### 2.2.1 Within upland nesting habitat, what is the proximity of bird nests and predator locations to specific habitat features?

To determine how nests and predators within the core upland nesting area were located relative to specific habitat features, including some habitat edge features such as roads/levees, canals, and wetland boundaries, we calculated the distances between those features and nest locations, predator GPS locations, and random locations, and then conducted the following statistical analyses.

##### Nest proximity to habitat features

We included nests of the three main monitored dabbling duck species (mallard, gadwall, and cinnamon teal, *Spatula cyanoptera*; hereafter duck nests) and the most common non‐waterfowl bird species we monitored (northern harrier), including nests that were discovered depredated (partially or completely) or already hatched. We associated each nest with the seasonal water layer from the year and month (April, May, or July) that best corresponded to when the nest was monitored.

##### Predator proximity to habitat features

We extracted a subset of raccoon and skunk locations that represented the use of the core upland duck nesting area. To do this, we used the boundary of the area searched for duck nests, buffered by an additional 25 m (8.5 km^2^ total area; Figure 1). Each predator GPS location was associated with the seasonal water layer from the year and month (April, May, or July) that was closest in time to the location. Raccoons from 2016 were excluded from the analysis of distance to ATV paths because the satellite imagery was poor for 2016 and ATV paths could not be accurately digitized. For each collared individual, we also quantified the percent of locations for the duration of the duck nesting season that fell within the core upland nesting area.

##### Random locations in proximity to habitat features

To describe if real duck nests, northern harrier nests, and collared animals were located closer to or further from each of the 10 habitat features than would be expected by chance, we placed random points on the landscape within the core upland nesting area. A random point could be located anywhere within the same boundaries that we used to identify upland‐associated predator locations (Figure 1). We selected the same number of random locations as monitored duck nests.

##### Statistical analysis

All statistical analyses were conducted in the program R v. 4.0.5. (R Core Team, 2020). For this analysis, we calculated the distance between each nest location, collared animal location, or random location and the closest polygon or polyline within each type of habitat feature by using the *gDistance* function from the *rgeos* package (Bivand & Rundel, 2020). For each habitat feature, we conducted a separate mixed effects linear model (Bates et al., 2015). Distance to the habitat feature was the response variable with location type as a categorical fixed factor (random location, duck nest location, harrier nest location, female raccoon location, male raccoon location, female skunk location, and male skunk location), and collared animal identification was included as a random factor to account for repeated measures of collared predators. Significance was determined with *F* tests from the *afex* R package, using Satterthwaite approximation for degrees of freedom (Singmann et al., 2015). Post‐hoc pairwise tests on least squares mean distances were conducted to determine differences in the mean distance to each habitat feature among the seven levels. Models were analyzed using log_e_ transformed distances because the residuals from models with untransformed data were not normally distributed; half of the minimum non‐zero distance for each habitat feature that included 0s was added to each distance prior to the log_e_ transformation. We report back‐transformed least squares mean distances and 95 percent confidence intervals.

#### 2.2.2 Is the probability of nest predation correlated with distance to habitat features?

We used a logistic exposure method to model the daily probability of a nest ‘surviving’ (not discovered and depredated) within a standard nest survival framework (Shaffer, 2004) and tested if the probability of a nest surviving was influenced by the proximity of specific habitat features at the landscape scale. In this analysis, there was no knowledge of the predator species responsible for nest predation. Probabilities were modeled as a function of categorical, continuous, and time‐specific predictor variables. Each individual nest remained in the analysis until the first nest monitoring visit with evidence of predation (partial or complete predation), irrespective of whether the nest was active (hen still present) or already inactive at that point, since inactive nests can still contribute food resources to a predator. A nest (of any status) was considered to have survived the interval if it was not discovered and not depredated by a predator. The nest age associated with each interval between nest monitoring visits was the estimated age at the start of each interval.

We tested models hierarchically by first testing all combinations of a suite of variables known to influence nest survival, including year, species, nest initiation date (linear and quadratic terms), nest age (linear and quadratic terms), nest status (active or inactive nest), and an interaction between nest status and nest age (with both linear and quadratic terms for day of year) (Pieron & Rohwer, 2010). Quadratic terms were included to test if nest survival had a quadratic shape with nest initiation date or nest age. The interaction between nest status and nest age was tested to determine if the probability of a nest surviving had a different relationship with nest age depending on whether the hen was still actively attending her nest or if eggs remained in an inactive nest. Models were ranked using second‐order Akaike Information Criterion (AIC_c_) in an information‐theoretic approach (Burnham & Anderson, 2002).

We determined that the best base model to use when quantifying nest survival as a function of distance to habitat features included year, species, nest status, nest age (quadratic), and an interaction between nest status and nest age (quadratic; Table 2). Once we determined the best base model structure, we then tested the base model with a full set of additional models that included all combinations of the distances or log_e_‐transformed distances to nine habitat features described above as well as the distance to the nearest drivable levee/road (a subset of levee/road) and the nearest vegetation patch (a combination of phragmites and shrub patches). To avoid the inclusion of redundant habitat features, we did not allow the same model to include the following combinations: (1) canals and drivable levee/road (because canals were often bordering drivable levees/roads), (2) vegetation and either phragmites or shrub, (3) drivable levee/road and levee/road and (4) untransformed and log_e_‐transformed distance to the same habitat feature. No remaining pairs of habitat features were strongly correlated (all correlation coefficients ≤0.65). Half of the minimum non‐zero distance for each habitat feature that included 0s (e.g., a nest within a vegetation patch) was added to each distance prior to the log_e_ transformation.

**TABLE 2:** Model selection results to determine the base model for examining the probability of mallard (*Anas platyrhynchos*), gadwall (*Mareca strepera*), or cinnamon teal (*Spatula cyanoptera*) nest ‘survival’ (not being discovered and depredated) relative to different habitat features at the Grizzly Island Wildlife Area, Suisun Marsh, California, 2016–2019.

Model	*k*	−2LogL	AIC_c_	ΔAIC_c_	*w*_*i*_	Evidence ratio
**year + species + nest status + nest age** ^**2**^ **+ nest status × nest age** ^**2**^	**11**	**5004.65**	**5026.65**	**0.00**	**0.57**	**1.00**
year + species + nest status + nest age^2^ + nest status × nest age^2^ + initiation date	12	5003.62	5027.63	0.98	0.35	1.63
**year + species + nest status + nest age** ^**2**^ **+ nest status × nest age**	**10**	**5012.89**	**5032.89**	**6.24**	**0.03**	**22.65**
**year + species + nest status + nest age + nest status × nest age**	**9**	**5017.28**	**5035.29**	**8.63**	**0.01**	**74.98**
year + nest status + nest age^2^ + nest status × nest age^2^	9	5021.74	5039.75	13.09	<0.01	696.83
**year + species + nest status**	**7**	**5051.52**	**5065.53**	**38.87**	**<0.01**	**2.76 × 10** ^**8**^
**year + nest status + nest age** ^**2**^	**7**	**5064.16**	**5078.16**	**51.51**	**<0.01**	**1.53 × 10** ^**11**^
**year + species + nest status + nest age** ^**2**^	**9**	**5046.82**	**5064.82**	**38.17**	**<0.01**	**1.94 × 10** ^**8**^
**year + species + nest age** ^**2**^	**8**	**5059.85**	**5075.85**	**49.20**	**<0.01**	**4.83 × 10** ^**10**^
**species + nest status + nest age** ^**2**^ **+ nest status × nest age** ^**2**^	**8**	**5129.10**	**5145.10**	**118.45**	**<0.01**	**5.26 × 10** ^**25**^
*null model*	1	5235.94	5237.94	211.29	<0.01	7.59 × 10^45^

*Note*: Models in the table represent all models within a ΔAIC_c_ of 4 from the top model as well as all models with a single factor from the top model removed. The following terms are reported for each model (and all model selection tables in the paper): *k* (number of parameters in the model), −2LogL (−2 × log[likelihood]), AIC_c_ (second‐order Akaike information criterion), ΔAIC_c_ (the difference in the AIC_c_ between the top model and the model of interest), *w*_*i*_ (Akaike model weight), evidence ratio (how many times more likely the top model is over the model of interest). Bolded models are the top model and those that are the same as the top model, but have a single variable removed.

After running a full model set with all combinations of variables (*n =* 5036 models), we determined that most untransformed distances to habitat features performed better than transformed data, except for the distance to seasonal wetlands, and separate variables for phragmites and shrub patches performed better than a combined vegetation variable. Furthermore, the distance to levees/roads was better supported as a potential predictor variable than the distance to a drivable road levee when canals and drivable levees/roads were not allowed in the same model. Thus, our final and balanced model set included the variables in the base model, the untransformed distance to the nearest: ATV path, levee/road, canal, shrub patch, phragmites patch, tree, telephone pole, and human structure, and the log_e_‐transformed distance to the nearest seasonal wetland (*n* = 512 total models; *n* = 1618 nests; Peterson & Ackerman, 2022).

We set the maximum number of terms in the final set of models at 16, which allowed for all habitat features in the final model set to be included in the same model in addition to the variables in the base model but still avoided over‐parameterization. Unless otherwise specified, to account for model selection uncertainty, we generated model‐averaged predictions, and plotted predictions for mallards at active nests during 2018 at the median value for non‐focal continuous variables. Model averaged predictions were calculated by averaging the predicted values that were generated from each model individually within the candidate model set using the coefficients from that model and weighting the contribution of the predictions from each model by the model weight. Model averaged predictions were not generated by using model‐averaged coefficients. We evaluated support for individual variables based on the 85% confidence interval around conditional model‐averaged coefficients (Arnold, 2010). We estimated the cumulative probability of surviving to 35 days (expected time interval from nest initiation to hatch) as the product of model‐averaged daily survival rates and we used the delta method to obtain the standard error for cumulative survival rates (Powell, 2007; Seber, 1982) and determine 95% confidence intervals.

#### 2.2.3 How do raccoons and skunks encounter bird nests as they move each night?

##### Distance traveled per night of foraging

To estimate the approximate distance that collared predators typically move during a night of foraging, we quantified how far raccoons and skunks traveled per night during duck nesting. We ran a generalized additive mixed effects model (GAMM) with the *gam* function from the *mgcv* package (Pedersen et al., 2019), to account for the potential influence of date on nightly movements without forcing the model to adhere to a specific polynomial function (e.g., linear, quadratic, or cubic). We included an individual smoother for each factor level in a four‐factor species × sex term and allowed for separate intercepts for each factor level, with a random effect of individual animal identification: distance_per_night ~ s(julian_day, by = species_sex_factor, bs = “tp”) + s(animal_identification, bs = “re”) + species_sex_factor. The model was run using default parameters for the link function (‘identity’) and the smoothing basis dimension (*k*), and we used restricted maximum likelihood (REML) to estimate model coefficients and smoothing parameters. We extracted model‐estimated predictions for the distance traveled per night at key time periods during duck nesting, specifically the onset of duck nesting (5% of monitored duck nests had been initiated) and the midpoint of duck nesting (50% of monitored duck nests had been initiated).

##### Nests within individual home ranges and encounters by collared predators

To contextualize the range of potential influence of individual collared predators on bird nests, we calculated the number of monitored bird nests within the outer boundaries of each individual collared predator. For this, we conducted fixed kernel density estimates using the *ks* package (Duong et al., 2019), using a bivariate kernel and the plug‐in method of bandwidth selection. We extracted the 99th percentile contour, removed any holes within the 99th percentile contour, and buffered the edge of the contour by 100 m, which provided a smoothed outer boundary that we used to identify nests that could have been encountered by each collared individual. We buffered the outer boundary of the kernel by 100 m to account for some encountered nests that we documented to occur at the edge of individual home ranges and would otherwise have been excluded as an available nest. This metric of space use was not intended to quantify the absolute home range size or determine where individuals spent most of their time but as a tool to identify available nests for each collared animal. Not all animals were tracked for the same length of time; thus, we examined the percent of nests that were encountered by each individual out of the total number of available nests that had eggs present while the animal was being tracked. We included all 41 collared individuals when we estimated the minimum number of nests for a season within the outer boundaries of each individual home range.

**FIGURE 2:**
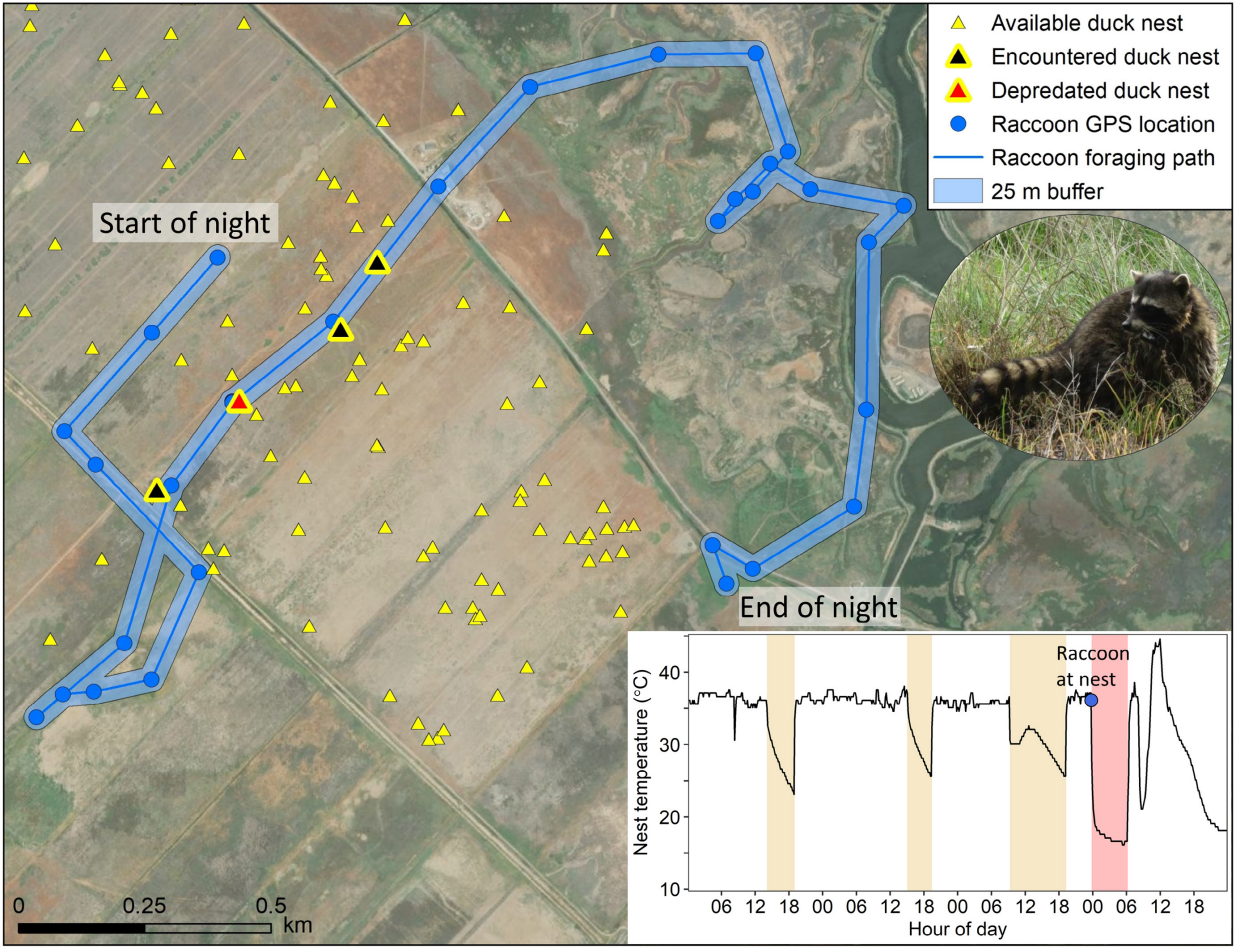
An example of one night of GPS locations from a collared raccoon (*Procyon lotor*), showing GPS locations collected every 15 min, the putative path of the animal buffered by 25 m, monitored bird nests (mostly duck nests), bird nests within the buffered movement path (encountered nests) and a duck nest with evidence of predation at the subsequent nest monitoring visit. The inset figure shows the nest temperature record for the depredated nest during the 3 days prior to the encounter and all the way through the predation of the nest by the collared raccoon. Diurnal incubation recesses, when the duck hen voluntarily left her nest during the day, are shown in tan and the nocturnal recess, when the raccoon flushed the hen off the nest and the eggs were left uncovered, is shown in red.

We used GPS locations to examine encounters with monitored bird nests on a nightly basis for 24 adult raccoons (*n =* 10 male, *n =* 14 female) and 13 adult skunks (*n =* 6 male, *n =* 7 female), excluding the other four collared individuals from this analysis because those collar deployments did not last for more than a week of nest monitoring (monitoring failed ~April 1). Encounters were initially evaluated with buffer distances of 10, 25, and 50 m and the results were compared; 10 m was an overly conservative buffer and underestimated nest encounters when we had subsequent evidence of depredation at the nest whereas 50 m did not add many additional nest encounters with evidence of depredation and may have been outside of the distance for predator detectability (Nams, 1997). Thus, based on the temporal frequency of GPS locations, the possible error in the GPS location, and the scale of predator detection, we selected 25 m as the most biologically supported distance to evaluate nest encounters and nest discovery. For each animal, sequential locations for each night were converted to a line and the putative animal path was buffered by 25 m; all monitored nests that fell within the buffer were considered nest encounters (Figure 2). Additionally, each nest encounter was categorized as a predation encounter if the nest had evidence of predation at the nest monitoring visit after the nest encounter, indicating that the predator could have been responsible for damaging or consuming at least one egg at the nest. If all the eggs were missing or destroyed at the nest visit, the nest was considered completely depredated. The nest was considered partially depredated if at least one whole egg remained undamaged in the nest (Ackerman et al., 2003). We then quantified the average nightly rate of nest encounters and encounters with depredated nests per individual predator during the central span of nest monitoring from April 17 to June 14 (10th–90th quantiles of nest monitoring dates).

##### Evidence of collared predators discovering encountered nests when hens were present

We used a subset of active duck nests (hen still attending to eggs) to gain deeper insight into the steps between a collared predator walking within 25 m of an active nest (nest encounter) and the predator actually locating and depredating the nest, as well as the subsequent consequences for the fate of the nest. We quantified the proportion of nest encounters where the proximity of a collared predator ≤25 m of a nest coincided with an identified incubation recess (within 30 min) and if the subsequent nest monitoring visit revealed evidence of predation at the nest (Figure 2). Furthermore, using the rate of temperature decrease (Croston et al., 2021), we examined if the hen covered her eggs or left her eggs uncovered when she left the nest and whether that affected the likelihood of the predator locating and depredating the nest. Additionally, if the final hen's departure from the nest was within 30 min of the predator encountering the nest, the predator encounter was categorized as causing nest abandonment, regardless of whether the nest was fully depredated or if the nest did not have visual evidence of predation at the next investigator visit.

#### 2.2.4 Are predator‐specific nest encounter probabilities influenced by the proximity to habitat features?

We conducted predator‐specific analyses to determine if the probability of a nest (including both active and inactive nests) remaining unencountered by a collared predator was influenced by the proximity to habitat features. For this analysis, we selected individual raccoons (*n =* 7) and skunks (*n =* 5) that demonstrated substantial use of upland habitat and encountered ≥10 duck nests. We extracted all nests within the home ranges of each collared predator and used a logistic exposure method, within a standard nest survival framework, to estimate the probability of a nest remaining unencountered by a collared predator as a function of distance to habitat features, with each night as a separate exposure period for every nest. Each individual nest remained in the analysis until the first night when the nest was encountered by a collared predator. Models were run separately for each predator species. In this analysis, we defined nest encounters as a collared predator of that species being within 25 m of a nest, irrespective of whether the nest had evidence of predation. Thus, this analysis does not show true predation but suggests the vulnerability of an individual nest to predation, with the assumption that predators first must come close to a nest to have a chance to find it and depredate eggs. We followed a similar process as described earlier in the methods (Section 2), although we reduced the base model to only include nest age (quadratic and linear terms), removing species, year, and nest status from this model. We used the same final model set of habitat features as described earlier, although we removed ATV path as a habitat feature because one of these raccoons was collared in 2016 when we could not digitize ATV paths.

## 3 RESULTS

We monitored 2008 nests of nine ground‐nesting bird species from 2016 to 2019 (Figure 1); 608 nests in 2016, 468 nests in 2017, 301 nests in 2018, and 631 nests in 2019. Of these, three species of dabbling ducks (mallard: *n =* 1074, gadwall: *n =* 828, cinnamon teal: *n =* 64) comprised 97.9% of nests and the remaining 42 nests included northern harrier (*n =* 18), ring‐necked pheasant (*Phasianus colchicus*; *n =* 10), American bittern (*Botaurus lentiginosus*; *n =* 9), northern pintail (*Anas acuta*; *n =* 2), northern shoveler (*Spatula clypeata*; *n =* 2), and short‐eared owl (*Asio flammeus*; *n =* 1). For most analyses, we focused on the three main duck species; however, all monitored nests were used to examine collared predator movement in relation to known bird nests. Overall, 64.2% of monitored nests had evidence of predation at some point during nest monitoring (active and inactive nests), including 83 nests (4.1%) that were completely depredated at the time of discovery.

### 3.1 Within upland nesting habitat, what is the proximity of bird nests and predator locations to specific habitat features?

**FIGURE 3:**
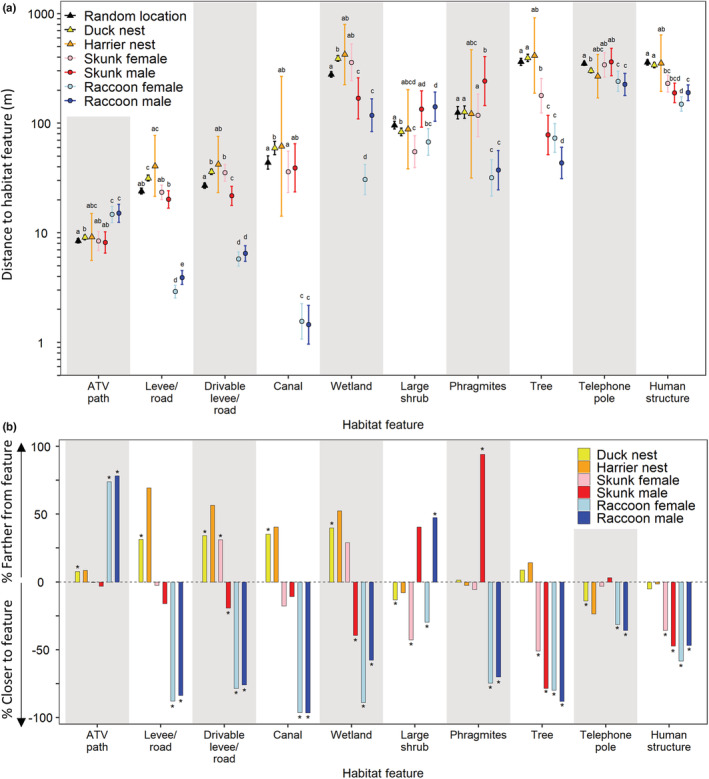
(a) Model‐generated mean distances (least squares mean with 95% CI) between 10 different habitat features and seven types of locations: Random locations, nest locations (duck nests and northern harrier nests), and predator locations (males and females of both predator species) within the core upland duck nesting area (models were conducted separately for each habitat feature). Within each habitat feature type, letters indicate significant differences (*p* < .05) between the types of locations. (b) The percent difference between nest or predator locations and random locations within each of the habitat features. Positive percent differences indicate that nests or predators were located further from the habitat feature than a random location and negative percent differences indicate that nests or predators were located closer to each habitat feature than a random location. Asterisks indicate significant differences from random locations.

Compared to predator locations within the core upland nesting habitat, duck nests tended to be located farther from several of the habitat features than raccoon locations but overlapped more in the distance to habitat features with skunk locations (Figure 3; Peterson & Ackerman, 2022). Specifically, duck nests were located ≥27.6 m farther than female and male raccoons from levees/roads, ≥57.7 m farther from canals, ≥61.2 m farther from telephone poles, ≥89.0 m farther from phragmites patches, ≥149.2 m farther from human structures, ≥271.4 m farther from seasonal wetlands, and ≥321.5 m farther from trees (all *t* ≥ 2.06, all *p* ≤ .040). Additionally, duck nests were located similar distances to shrub patches as female raccoons (*t* = 1.43, *p* = .15) but were 58.0 m closer to shrub patches than male raccoons (*t* = 3.29, *p* = .001). When compared to female and male skunks, duck nests were located ≥8.1 m farther from levees/roads, ≥216.5 m farther from trees, and ≥108.9 m farther from human structures (all *t* ≥ 3.58, all *p* ≤ .001), and were located similar distances from ATV paths and telephone poles (all *t* ≤ 1.22, all *p* ≥ .22). When compared to female skunks, nests were 23.2 m farther from canals and 28.4 m farther from shrub patches (all *t* ≥ 2.14, all *p* ≤ .033), whereas nests were located similar distances as female skunks from drivable levees/roads, seasonal wetlands, and phragmites (all *t* ≤ 0.41, all *p* ≥ .68). When compared to male skunks, nests were 51.3 m closer to shrub patches and 115.7 m closer to phragmites but were located 14.4 m farther from drivable levees/roads and 221.9 m farther from seasonal wetlands (all *t* ≥ 2.40, all *p* ≤ .017; Figure 3). Examining each habitat feature independently, nest locations and predator locations were closer and farther to some habitat features than expected by chance (Figure 3). In particular, duck nests, on average, were located farther from canals than by chance (*t* = 2.98, *p* = .003); female and male raccoons were located closer to canals than by chance (all *t* ≥ 15.62, all *p* ≤ .001); neither northern harrier nests nor skunks were located farther away, or closer to, ATV paths than by chance (all *t* ≤ 0.84, all *p* ≥ .40); and raccoons were located farther from ATV paths than by chance (all *t* ≥ 5.99, all *p* < .001).

During the duck nesting season, more than 50% of an individual collared raccoon or skunk's locations fell within the core upland nesting area for 8% of raccoons (2 of 25 raccoons) and 56% of skunks (9 of 16 skunks; Table 1). Individual female raccoons were located within the core upland nesting area 15.8 ± 18.2% (mean ± SD) of the time (range: 0.0%–52.1%) and male raccoons were located within the core upland nesting area 15.1 ± 17.2% of the time (0.0%–57.0%). Individual female skunks were located within the core upland nesting area 76.7 ± 16.6% of the time (45.0%–100.0%) and male skunks were located within the core upland nesting area 37.9 ± 32.0% of the time (0.3%–82.4%).

### 3.2 Is the probability of nest predation correlated with distance to habitat features?

**FIGURE 4:**
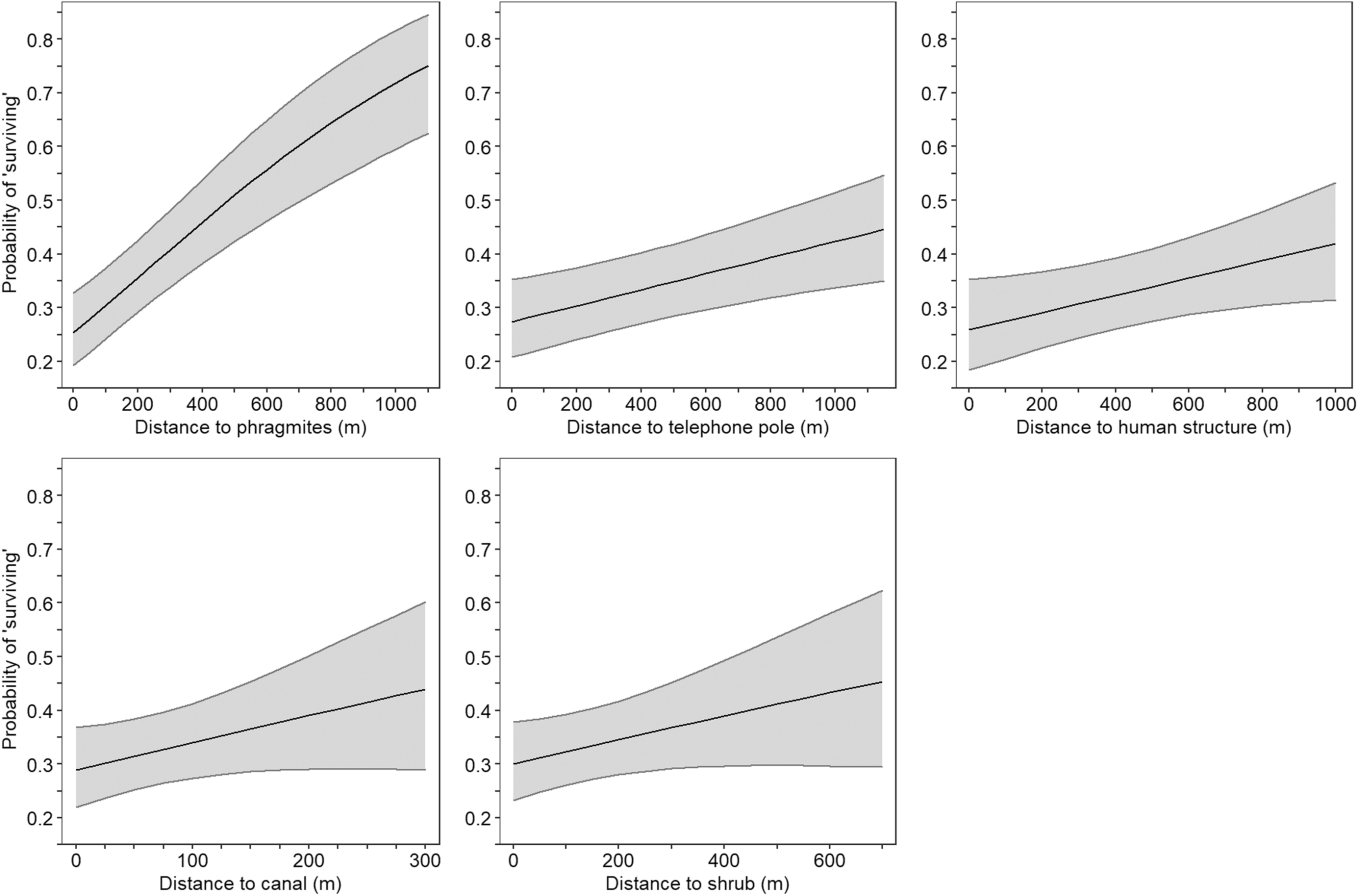
Predicted cumulative probabilities (with 95% CI obtained after using the delta method to estimate standard error) of an active mallard (*Anas platyrhynchos*) nest ‘surviving’ (not being discovered and depredated by any predator) over the 35‐day period from nest initiation to expected hatch, as a function of the distance to the nearest habitat feature for the features included in the top model. Model‐averaged predictions were generated for nests in 2018, holding the distance to additional habitat features at the median value and the cumulative probability was calculated as the product of daily survival estimates. The *x*‐axis represents the range of observed data for each habitat feature for all nests in the analysis (*n* = 1618 nests).

At the landscape scale, the probability that a nest would survive (not be discovered and depredated) increased with increasing distance to phragmites patches, shrub patches, human structures, telephone poles, and canals, after accounting for the variables included in the base model (Figure 4; Table 3). For the year 2018, when all other variables were held at their median value, the cumulative probability of an active mallard nest surviving for 35 days increased when a nest was located 500 m from the following habitat features when compared to being immediately adjacent to the feature: 25.4% (SE: 3.4%) to 51.0% (4.4%) for phragmites, 30.0% (3.7%) to 41.2% (6.2%) for shrubs, 25.9% (4.3%) to 33.9% (3.4%) for human structures, and 27.4% (3.7%) to 34.8% (3.4%) for telephone poles. Additionally, the cumulative probability of a nest surviving to 35 days increased from 28.8% (3.8%) when a nest was adjacent to a canal to 34.0% (3.5%) when a nest was 100 m from a canal (Figure 4). There were four additional models within a ΔAIC of 2 from the top model but each of these four models was identical to the top model with the addition of a single variable (either ATV paths, levees/roads, trees, or the log_e_‐transformed distance to seasonal wetlands), most of which were determined to be uninformative parameters (Table 3).

**TABLE 3:** Model selection results for a modified nest survival analysis on the probability of mallard (*Anas platyrhynchos*), gadwall (*Mareca strepera*), or cinnamon teal (*Spatula cyanoptera*) nest ‘survival’ (not being discovered and depredated) at the Grizzly Island Wildlife Area, Suisun Marsh, California, 2016–2019.

Model (base model: Year + species + nest status + nest age^2^ + nest status × nest age^2^)	*k*	−2LogL	AIC_c_	ΔAIC_c_	*w*_*i*_	Evidence ratio
**+ canal + human structures + phragmites + shrub + telephone pole**	**16**	**4919.79**	**4951.81**	**0.00**	**0.15**	**1.00**
+ canal + human structures + phragmites + shrub + telephone pole + atv	17	4918.88	4952.90	1.09	0.09	1.73
+ canal + human structures + phragmites + shrub + telephone pole + log(wetland)	17	4919.39	4953.41	1.60	0.07	2.23
+ canal + human structures + phragmites + shrub + telephone pole + tree	17	4919.68	4953.70	1.89	0.06	2.57
+ canal + human structures + phragmites + shrub + telephone pole + levee	17	4919.74	4953.76	1.95	0.06	2.66
+ canal + human structures + phragmites + shrub + telephone pole + atv + log(wetland)	18	4918.53	4954.55	2.74	0.04	3.93
+ canal + human structures + phragmites + shrub + telephone pole + atv + levee	18	4918.79	4954.81	3.00	0.03	4.48
+ canal + human structures + phragmites + shrub + telephone pole + atv + tree	18	4918.80	4954.82	3.01	0.03	4.51
**+ canal + human structures + phragmites + telephone pole**	**15**	**4925.24**	**4955.26**	**3.45**	**0.03**	**5.61**
+ canal + human structures + phragmites + shrub + telephone pole + log(wetland) + levee	18	4919.35	4955.37	3.56	0.03	5.94
+ canal + human structures + phragmites + shrub + telephone pole + log(wetland) + tree	18	4919.36	4955.38	3.57	0.03	5.97
**+ human structures + phragmites + shrub + telephone pole**	**15**	**4925.38**	**4955.39**	**3.58**	**0.03**	**6.00**
+ canal + human structures + phragmites + shrub + telephone pole + tree + levee	18	4919.64	4955.66	3.85	0.02	6.86
**+ canal + phragmites + shrub + telephone pole**	**15**	**4928.16**	**4958.18**	**6.37**	**0.01**	**24.15**
**+ canal + human structures + phragmites + shrub**	**15**	**4930.18**	**4960.19**	**8.38**	**<0.01**	**66.17**
**+ canal + human structures + shrub + telephone pole**	**15**	**4953.699**	**4983.714**	**31.91**	**<0.01**	**8.48 × 10** ^**6**^
*null (base) model*	11	5004.65	5026.65	74.85	<0.01	1.79 × 10^16^

*Note*: This analysis examined the probability of nest survival based on nest monitoring data and any evidence of predation at monitored nests. All models also include the same set of base variables: Year + species + nest status + nest age^2^ + nest status × nest age^2^ (Table 2). All variables represent the distance to that habitat feature from a nest; the distance to the nearest wetland was log_e_ transformed prior to analysis. Models in the table represent all models within a ΔAIC_c_ of 4 from the top model as well as all models with a single factor from the top model removed. Bolded models are the top model and those that are the same as the top model, but have a single variable removed.

### 3.3 How do raccoons and skunks encounter bird nests as they move each night?

#### 3.3.1 Distance traveled per night of foraging

**FIGURE 5:**
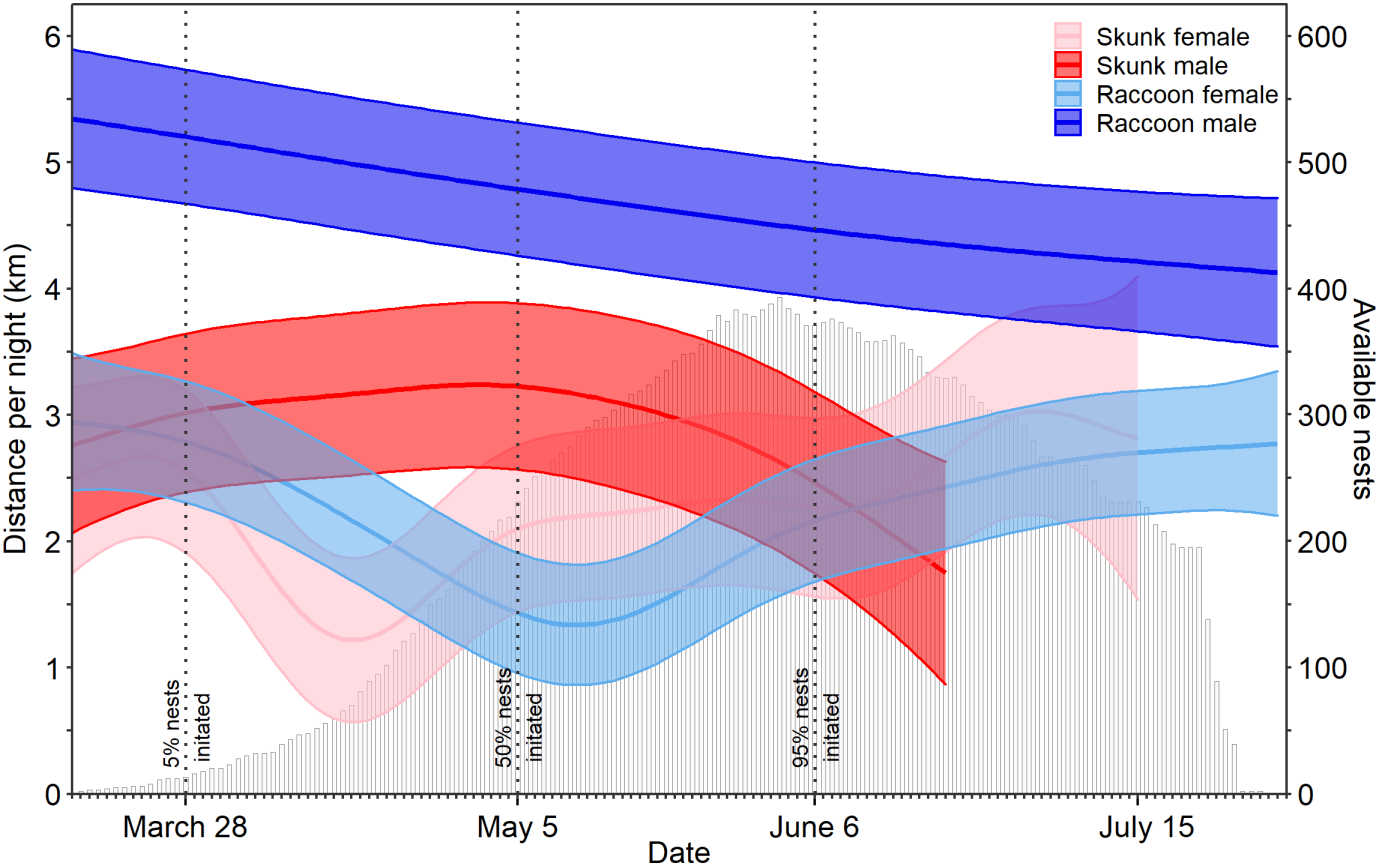
Model‐predicted mean (with 95% CI) nightly distance traveled by adult raccoon (*Procyon lotor*) and skunk (*Mephitis mephitis*) females and males during duck nesting in Suisun Marsh, California. Predictions from a generalized additive mixed effects model are only shown for the duration of the time that GPS locations were collected, and *x*‐axis tick marks are at a daily scale with key duck nesting time periods denoted. Female skunks and raccoons, in contrast with their male counterparts, both had a marked drop in the mean nightly distance traveled on approximately April 16 (Julian day 106) for skunks and approximately May 12 (Julian day 132) for raccoons, followed by a subsequent increase in the distances traveled each night late in duck nesting. Duck nests with eggs present (available resource for a predator) within the core upland nesting area are shown in gray bars (shown for duck nests monitored in 2019).

Over the duck nesting season, model‐estimated mean nightly distance traveled varied with date and was influenced by predator species and sex (Figure 5). When ducks began to nest (March 28; 5% of monitored duck nests in our study had been initiated), male raccoons traveled 5.20 km (SE: 0.27 km) each night, which was 86.4% farther than the 2.79 km (0.24 km) traveled nightly by female raccoons. Male skunks traveled 3.02 km (0.32 km) each night, which was 18.4% farther than the 2.55 km (0.33 km) traveled each night by female skunks. When approximately 50% of monitored nests from our study had been initiated (May 5), the nightly distance traveled by male raccoons, female raccoons, male skunks and female skunks was 4.79 km (0.27 km), 1.43 km (0.24 km), 3.22 km (0.34 km) and 2.10 km (0.34 km), respectively. The maximum calculated distance traveled over a given night during duck nesting ranged from 3.91 to 13.41 km for individual raccoons and 3.23 to 10.28 km for individual skunks.

#### 3.3.2 Nests within individual home ranges and encounters by collared predators

**FIGURE 6:**
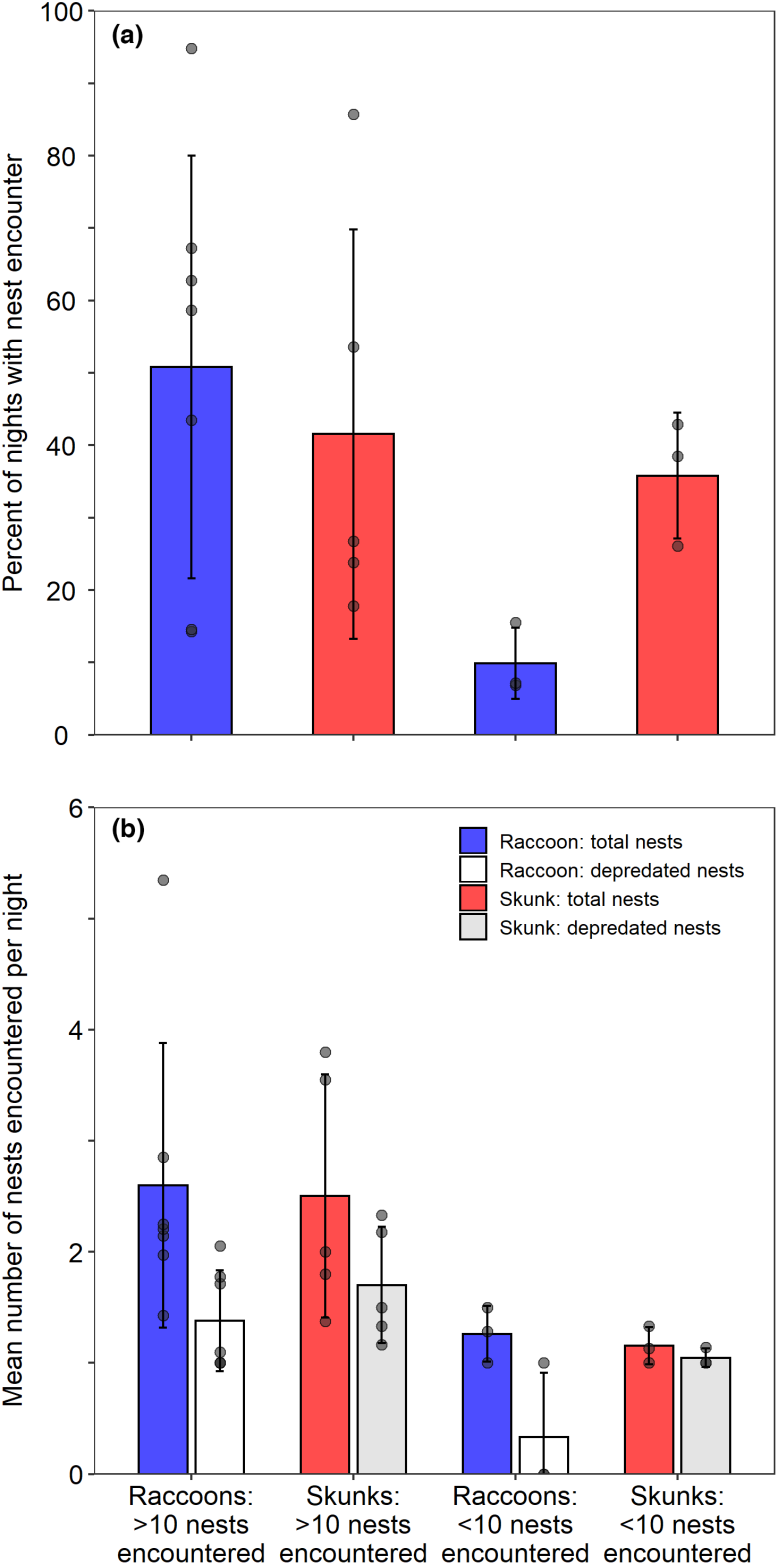
(a) Percent of nights during the central span of duck nesting (58 nights: April 17–June 14) that GPS‐collared raccoons (*Procyon lotor*) and striped skunks (*Mephitis mephitis*) came within 25 m of a monitored duck nest (nest encounter), excluding collared animals that never encountered >1 duck nest. Bars show arithmetic mean ± SD with individual values indicated by circles. Raccoons and striped skunks were presented separately based on whether each individual encountered more or fewer than 10 duck nests over the course of the nesting season, and this division was used to identify the collared animals that were used to examine how the probability of a nest encounter relates to the proximity to specific habitat features. (b) The mean number of total nests encountered per night, shown as the mean of individual averages, and the number of nests encountered per night with evidence of depredation (observed the subsequent nest monitoring visit). These averages are only calculated from nights when an individual encountered ≥1 nest. For visualization, SD was truncated at 0.

Of the 25 collared raccoons, 22 (88.0%) had at least one monitored bird nest (range: *n =* 2–294 nests) within their buffered home range when the predator was being tracked but only 12 raccoons (48.0%) encountered ≥1 nest within 25 m (*n =* 1–144 nests). For these 12 individuals, 19.3 ± 13.2% (mean ± SD; range: 2.9%–49.0%) of nests within their home range were encountered. The remaining 13 raccoons did not appear to come within 25 m of any monitored nest. Of the 16 collared skunks, 87.5% had at least one monitored nest within their buffered home range (*n =* 2–173 nests within a home range for a given year) when the collar was working (Table 1), and nine skunks encountered ≥1 nest (*n* = 1–63 nests). For these nine individuals, 26.1 ± 8.7% (range: 15.7%–40.5%) of nests within their home range were encountered. For collared predators that encountered >1 nest over the nesting season, a higher proportion of nests encountered by skunks (51.9 ± 26.6%; *n* = 8 skunks) had evidence of predation at the next nest monitoring visit than did nests encountered by raccoons (22.3 ± 17.1%; *n* = 10 raccoons). Of the 18 individuals that encountered >1 nest during the central span of duck nesting (April 17–June 14), raccoons encountered nests during 1.7%–94.8% of nights and skunks encountered nests during 17.8%–85.7% of nights. The mean number of encountered and depredated nests are shown for the 18 skunks and raccoons separately by species based on whether each individual predator encountered more or fewer than 10 nests (Figure 6).

#### 3.3.3 Evidence of collared predators discovering encountered nests when hens were present

When a collared raccoon or skunk came within 25 m of a duck nest with the hen present, the predator did not discover and did not depredate the nest (partial or complete depredation) 96.2% of the time. However, when an active nest was discovered by a collared predator, it resulted in immediate nest failure (depredation and/or abandonment) more often when the predator was a raccoon than when it was a skunk. Hens remained at the nest 95.3% of the time when a raccoon encountered an active nest (of 464 total nest encounters by 12 raccoons) and 94.5% of the time when a skunk encountered an active nest (of 164 total nest encounters by 9 skunks). When hens left the nest in response to a nearby collared raccoon (*n =* 21 encounters at 20 nests by 4 collared raccoons), she left her eggs uncovered 85.7% of the time and the raccoon discovered and depredated eggs at 88.2% of those nests (one nest encounter was missing ambient temperature data and we could not predict if the hen covered the eggs when she left but the nest was discovered and depredated). The encounters where the hen covered her eggs before leaving the nest (*n* = 2) had no evidence of predation, suggesting the hen left the nest because of the predator but the predator never located the nest; in one case the hen returned and all 10 of her eggs subsequently hatched but in the second case the hen abandoned her nest. For 15 of the 20 nests where hens left the nest in response to a raccoon, it was their final departure from the nest and resulted in complete clutch depredation or abandonment. At one additional nest (not included in earlier summary calculations), a collared raccoon flushed the hen and her recently hatched chicks off the nest and depredated at least some of the brood. When hens left the nest in response to a nearby collared skunk (*n =* 9 encounters at 9 nests by 7 collared skunks), the hen left her eggs uncovered for 55.6% of nest encounters and the skunk discovered and depredated eggs at each of those nests. When the hen covered her eggs and left the nest (*n* = 4 encounters), the nest remained undiscovered by the collared skunk 75% of the time. For two of the nine nests, the hen never resumed incubation and both nests were completely depredated by the subsequent nest visit. In all other cases, the hen returned to the nest and resumed incubation.

### 3.4 Are predator‐specific nest encounter probabilities influenced by the proximity to habitat features?

**FIGURE 7:**
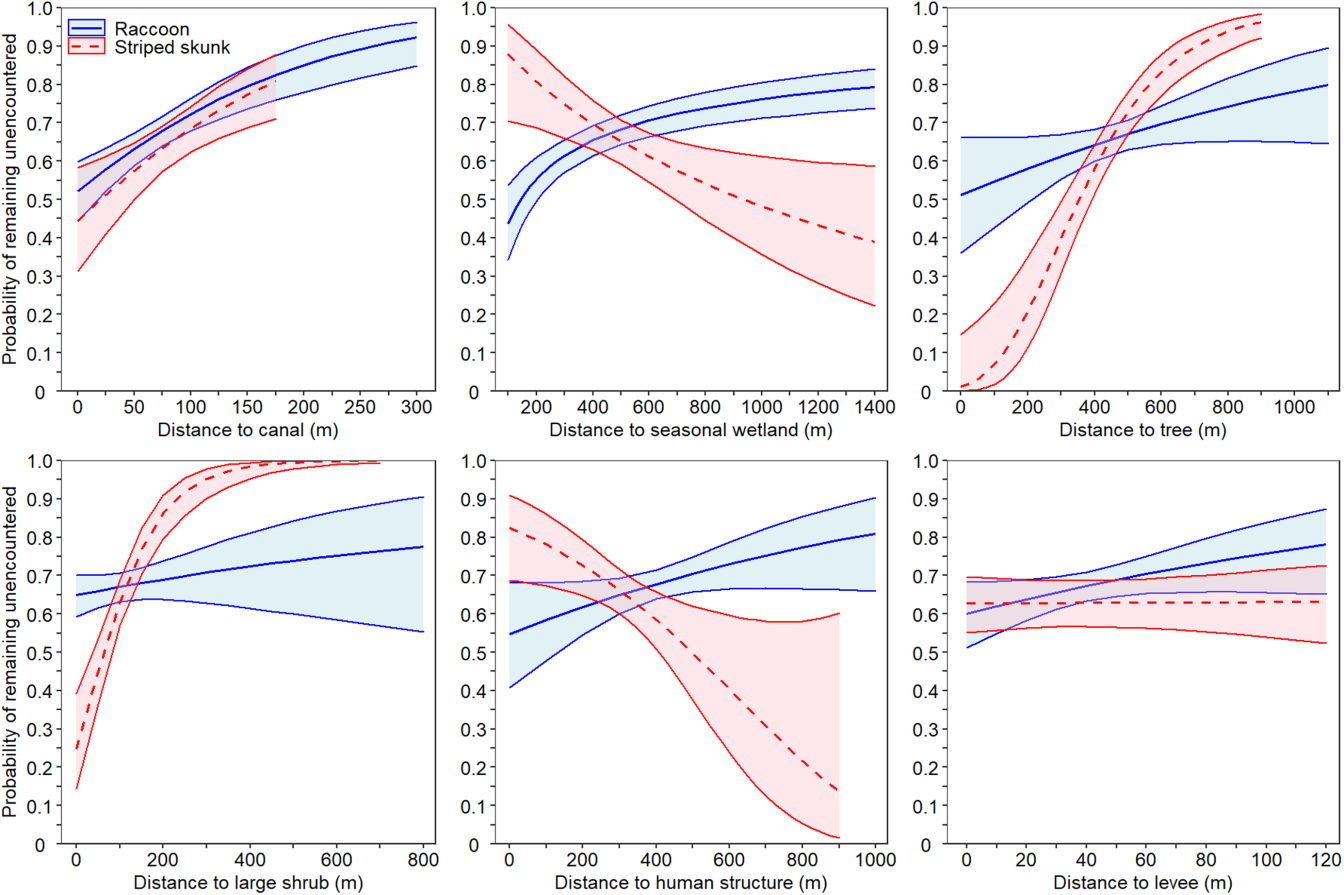
Predicted cumulative probabilities (with 95% CI obtained after using the delta method to estimate standard error) of a duck nest within the known home range of a collared predator remaining unencountered (GPS‐collared predator not observed ≤25 m from the nest) by a predator over a 35‐day period as a function of distance to different habitat features. Models were run separately for nests within the home ranges of GPS‐collared raccoons (*Procyon lotor*; *n* = 7 raccoons; *n* = 901 nests) and those within the home ranges of GPS‐collared skunks (*Mephitis mephitis*; *n* = 5 skunks; *n* = 345 nests). Model‐averaged predictions were generated for each habitat feature by holding the distance to additional habitat features at their median value and the cumulative probability was calculated as the product of daily survival estimates. The *x*‐axis represents the range of observed data for each habitat feature within this dataset and each predator species is only plotted to the extent of the data used in each model. Distance to telephone pole was also included in the top model for raccoons but is not shown here. Distance to levees/roads was not included in the top model for skunks but is shown here for comparison to raccoons. The distance to seasonal wetland was log_e_ transformed prior to statistical analysis.

Within the home ranges of collared raccoons, the probability of a nest remaining unencountered for 35 days increased with increasing distance to the nearest canal, seasonal wetland, tree, human structure, levee/road, telephone pole, and shrub patch (Figure 7; Table 4; *n* = 901 nests). When all other variables were held at their median value, the cumulative probability of a nest remaining unencountered by a collared raccoon for 35 days increased from 51.2% (SE: 7.9%) when a nest was immediately adjacent to a tree to 67.0% (2.0%) when a nest was located 500 m from a tree, from 54.8% (7.1%) when a nest was immediately adjacent to a human structure to 70.5% (2.3%) when a nest was located 500 m from a human structure, from 64.9% (2.8%) when a nest was immediately adjacent to a shrub patch to 73.9% (6.3%) when a nest was located 500 m from a shrub patch, from 64.2% (3.5%) when a nest was immediately adjacent to a telephone pole to 68.4% (2.0%) when a nest was located 500 m from a telephone pole, and from 43.7% (5.0%) when a nest was 100 m from a seasonal wetland to 68.4% (2.0%) when a nest was 500 m from a seasonal wetland. The probability of remaining unencountered by a collared raccoon increased from 52.2% (4.0%) when a nest was immediately adjacent to a canal to 72.3% (2.2%) when a nest was 100 m from a canal and from 60.0% (4.4%) when a nest was immediately adjacent to a levee/road to 75.8% (4.7%) when a nest was 100 m from a levee/road. No additional variables were supported (Table 4).

**TABLE 4:** Model selection results for a modified nest survival analysis on the probability of a mallard (*Anas platyrhynchos*), gadwall (*Mareca strepera*), or cinnamon teal (*Spatula cyanoptera*) nest remaining unencountered at a distance of 25 m by a GPS collared raccoon (*Procyon lotor*) at the Grizzly Island Wildlife Area, Suisun Marsh, California, 2016–2019.

Model (base model: Nest age^2^)	*k*	−2LogL	AIC_c_	ΔAIC_c_	*w*_*i*_	Evidence ratio
**+ levee + canal + human structure + log(wetland) + tree + shrub + telephone pole**	**10**	**2923.08**	**2943.09**	**0.00**	**0.20**	**1.00**
+ levee + canal + human structure + log(wetland) + tree + phragmites + telephone pole	10	2923.73	2943.74	0.65	0.14	1.38
+ levee + canal + human structure + log(wetland) + tree + shrub + phragmites + telephone pole	11	2921.90	2943.91	0.82	0.13	1.51
**+ levee + canal + human structure + log(wetland) + tree + shrub**	**9**	**2926.30**	**2944.31**	**1.22**	**0.11**	**1.84**
+ levee + canal + human structure + log(wetland) + tree + shrub + phragmites	10	2925.51	2945.52	2.43	0.06	3.36
+ levee + canal + human structure + log(wetland) + tree + phragmites	9	2927.82	2945.83	2.73	0.05	3.92
**+ levee + canal + human structure + log(wetland) + tree + telephone pole**	**9**	**2928.61**	**2946.62**	**3.53**	**0.03**	**5.84**
**+ canal + human structure + log(wetland) + tree + shrub + telephone pole**	**9**	**2929.03**	**2947.03**	**3.94**	**0.03**	**7.18**
**+ levee + canal + human structure + log(wetland) + shrub + telephone pole**	**9**	**2929.33**	**2947.34**	**4.24**	**0.02**	**8.35**
**+ levee + canal + log(wetland) + tree + shrub + telephone pole**	**9**	**2930.65**	**2948.65**	**5.56**	**0.01**	**16.12**
**+ levee + human structure + log(wetland) + tree + shrub + telephone pole**	**9**	**2949.82**	**2967.82**	**24.73**	**<0.01**	**2.35 × 10** ^**5**^
**+ levee + canal + human structure + tree + shrub + telephone pole**	**9**	**2950.27**	**2968.28**	**25.19**	**<0.01**	**2.95 × 10** ^**5**^
*null (base) model*	3	3063.84	3069.84	126.75	<0.01	3.34 × 10^27^

*Note*: All models include the same set of base variables: Nest age + nest age^2^ (quadratic). All variables represent the distance to that habitat feature from a nest; the distance to the nearest wetland was log_e_ transformed prior to analysis. Models in the table represent all models within a ΔAIC_c_ of 4 from the top model as well as all models with a single factor from the top model removed. Bolded models are the top model and those that are the same as the top model, but have a single variable removed.

Within the home ranges of collared skunks, the probability of a nest remaining unencountered for 35 days increased with increasing distance to the nearest tree, shrub patch, and canal and decreased with increasing distance to human structures and wetlands (Figure 7; Table 5; *n* = 345 nests). When all other variables were held at their median value, the probability of a duck nest remaining unencountered by a collared skunk increased from 1.2% (SE: 1.6%) when a nest was adjacent to a tree to 72.8% (2.9%) when a nest was 500 m from a tree and increased from 24.7% (6.4%) when a nest was adjacent to a shrub to 99.5% (0.4%) when a nest was 500 m from a shrub. Furthermore, the probability of remaining unencountered increased from 44.5% (7.1%) when a nest was adjacent to a canal to 68.6% (3.0%) when a nest was 100 m from a canal. Additionally, for these skunks utilizing the core upland nesting area, nests that were located closer to seasonal wetlands and human structures, which were on the outer edges of the core upland duck nesting area, were less likely to be encountered by collared skunks than nests located further from these habitat features (Figure 7). The probability of a nest remaining unencountered for 35 days increased from 65.2% (3.0%) when a skunk was 500 m from a wetland to 87.9% (6.0%) when a skunk was 100 m from a wetland. There was no support for any other variables (Table 5).

**TABLE 5:** Model selection results for a modified nest survival analysis on the probability of a mallard (*Anas platyrhynchos*), gadwall (*Mareca strepera*), or cinnamon teal (*Spatula cyanoptera*) nest remaining unencountered at a distance of 25 m by a GPS collared skunk (*Mephitis mephitis*) at the Grizzly Island Wildlife Area, Suisun Marsh, California, 2016–2019.

Model (base model: Nest age^2^)	*k*	−2LogL	AIC_c_	ΔAIC_c_	*w*_*i*_	Evidence ratio
**+ canal + human structure + log(wetland) + tree + shrub**	**8**	**1208.55**	**1224.57**	**0.00**	**0.25**	**1.00**
+ canal + human structure + log(wetland) + tree + shrub + telephone pole	9	1207.24	1225.25	0.69	0.18	1.41
+ canal + human structure + log(wetland) + tree + shrub + phragmites	9	1207.42	1225.44	0.87	0.16	1.55
+ canal + human structure + log(wetland) + tree + shrub + telephone pole + phragmites	10	1206.22	1226.24	1.67	0.11	2.31
+ canal + human structure + log(wetland) + tree + shrub + levee	9	1208.55	1226.57	2.00	0.09	2.71
+ canal + human structure + log(wetland) + tree + shrub + telephone pole + levee	10	1207.23	1227.25	2.68	0.07	3.82
+ canal + human structure + log(wetland) + tree + shrub + phragmites + levee	10	1207.41	1227.43	2.86	0.06	4.18
**+ canal + log(wetland) + tree + shrub**	**7**	**1219.27**	**1233.28**	**8.71**	**<0.01**	**77.98**
**+ canal + human structure + tree + shrub**	**7**	**1219.49**	**1233.50**	**8.93**	**<0.01**	**87.06**
**+ human structure + log(wetland) + tree + shrub**	**7**	**1223.26**	**1237.27**	**12.71**	**<0.01**	**574.03**
**+ canal + human structure + log(wetland) + shrub**	**7**	**1266.88**	**1280.89**	**56.32**	**<0.01**	**1.70 × 10** ^**12**^
**+ canal + human structure + log(wetland) + tree**	**7**	**1278.07**	**1292.08**	**67.51**	**<0.01**	**4.57 × 10** ^**14**^
*null (base) model*	3	1324.31	1330.31	105.74	<0.01	9.14 × 10^22^

*Note*: All models include the same set of base variables: Nest age + nest age^2^ (quadratic). All variables represent the distance to that habitat feature from a nest; the distance to the nearest wetland was log_e_ transformed prior to analysis. Models in the table represent all models within a ΔAIC_c_ of 4 from the top model as well as all models with a single factor from the top model removed. Bolded models are the top model and those that are the same as the top model, but have a single variable removed.

## 4 DISCUSSION

At the landscape scale, without differentiating species‐specific predator behaviors, we found that the farther duck nests were located from phragmites patches, large shrubs, telephone poles, other human structures, and canals, the less vulnerable they were to predation. The pronounced effect of distance to phragmites and shrub patches on nest survival may have been due to these habitats providing denning and diurnal resting sites for mammalian predators. The lack of a relationship between nest survival and the distance to other habitat features, including levees/roads, wetland boundaries, and trees, and the weak effect of distance to canals, was likely attributed to differences in species‐specific predator behaviors and distributions (Lahti, 2001; Raquel et al., 2015). For example, diurnal avian predators, such as common raven (*Corvus corax*), can complicate interpretation of nest predation at the landscape scale, as they perch on telephone poles and generally use habitats differently than mammalian predators (DeGregorio et al., 2014), even though they depredate eggs at a lower rate than mammalian predators (Croston, Ackerman, et al., 2018). For mammalian predators, different habitat features were important in explaining the probability of a nest being encountered by collared mammalian predators than the habitat features that were important in the probability of a nest surviving at the landscape scale. Within known home ranges of collared raccoons, the farther duck nests were located from canals, seasonal wetlands, trees, levees/roads, human structures, shrub patches, and telephone poles, the less likely they were to be encountered (predator ≤25 m of a nest). Within known home ranges of collared skunks, the farther duck nests were located from canals, trees, and shrub patches, the less likely they were to be encountered. However, nests that were located farther from seasonal wetlands and human structures were more likely to be encountered by a skunk, likely due to the high use of the interior of the upland nesting fields by skunks.

Our data suggest that there was not a strong response by predators to aggregate in the core upland nesting area during the seasonal pulse of available duck eggs that occurs in the nesting season, similar to Urban (1970). This result is consistent with the lack of landscape scale density‐dependent predation on duck nests at this study site (Ackerman et al., 2004; Ringelman et al., 2014). During the duck nesting season when eggs were available as an ephemeral prey resource, individual GPS‐collared raccoons and striped skunks varied widely in their use of the core upland nesting area. Numerous tagged raccoons and striped skunks in the study maintained home ranges adjacent to the core upland nesting habitat and did not aggregate within the upland nesting area to take advantage of the seasonally available prey resource, potentially because of either a general lack of interest, they were unaware of the availability of eggs as a resource, or, in the case of raccoons, they were repelled by territoriality (Chamberlain & Leopold, 2002; Pitt et al., 2008; Wehtje & Gompper, 2011). Instead, we found that only a small number of collared individuals (29% of marked raccoons and 39% of marked skunks) were responsible for 96% of the total nest encounters, similar to observations by Fritzell (1978). Raccoons that encountered the most duck nests had home ranges that included core upland nesting areas prior to the onset of duck nesting and maintained those home ranges throughout duck nesting. In each year, the outer boundary of a single raccoon home range contained 41% (2016: 250 nests), 63% (2017: 294 nests), 48% (2018: 145 nests), and 36% (2019: 229 nests) of all monitored nests. The level of territoriality demonstrated by raccoons varies among studies and may be driven by population structure and resource availability (Chamberlain & Leopold, 2002; Fritzell, 1978; Pitt et al., 2008; Prange et al., 2011; Wehtje & Gompper, 2011). If conspecific home ranges have relatively low overlap in this system, particularly for male raccoons, then predation of duck nests could be the result of individual specialization and opportunity within these predator populations. Thus, dominant male raccoons with stable boundaries could reduce the movements of other male raccoons and decrease access to duck eggs as a resource, similar to the protective territoriality observed in coyotes (*Canis latrans*; Sacks et al., 1999).

Within the core upland nesting area, raccoons and striped skunks demonstrated marked differences in how far they were located from certain habitat features, underscoring the importance of evaluating nest predation risk in the context of species‐specific and sex‐specific predator movements. Within the core upland nesting area, both male and female raccoons were located much closer to aquatic habitats, trees, phragmites patches, and levees and roads than female and male skunks and more than 57% closer to these habitat features than expected by chance. On average, skunks were located more than five times farther than raccoons from levees and roads, which appeared to be a commonly used travel corridor within the core upland nesting area for many collared raccoons (Figure 1). Raccoons did not appear to commonly use ATV paths within the upland fields as travel corridors, because raccoons were located more than 74% farther from ATV paths than expected by chance and were located more than 84% closer to levees/roads than expected by chance. Skunks, especially females, were more often located away from edge habitats such as levees/roads, canals, and wetland boundaries, and within the upland nesting fields (Ackerman, 2002). Although other studies observed high use of wetland habitat edges (Crabtree et al., 1989; Larivière & Messier, 2000), individual female skunks at this site were located farther from wetlands and human structures and within the core upland nesting area 77% of the time, whereas male skunks were only located within the core upland nesting area 38% of the time (Table 1). It is possible that female skunks were attracted to sites within the core upland nesting area because of available resting sites during the day and denning sites for pregnant females in phragmites patches. Rather than skunks being near to wetlands and human structures, which was a common observation in other studies, the large block of nesting fields may have provided habitat and foraging opportunities away from those features. In comparison, duck nests were located farther from habitat edges and potential travel corridors (e.g., levees/roads) and aquatic edge habitats (e.g., canals and seasonal wetlands) than would be expected by chance, which corresponded to a decreased likelihood that they would be encountered by raccoons and an increased likelihood that they may be encountered by skunks. In contrast, duck nests and northern harrier nests were not located farther from ATV paths than expected by chance (duck nests were 0.7 m farther from ATV paths but this was not a biologically meaningful distance). Based on our observations of individual movements and nest encounters (Table 1), female skunks may be a more significant nest predator than male skunks because of their smaller scale of movements within upland habitats and overlapping home ranges that provide for higher predator densities (Larivière & Messier, 2016).

Although both predator species are considered omnivorous with diverse diets, differences in association with wetland habitats near duck nests aligns with observations of raccoons avoiding upland grassland and crop fields and preferentially foraging along edge habitats such as forest, riparian, and wetland edges (Barding & Nelson, 2008; Cooper et al., 2015; Fritzell, 1978). Raccoons often show a preference for foraging on aquatic prey, especially crustaceans, when available (Rulison et al., 2012; Schoonover & Marshall, 1951; Urban, 1970). The observed proximity to habitat features like canals and travel corridors such as levees and roads suggests that raccoons did not typically move within the centers of upland nesting fields to search for duck nests, and that most raccoons likely preyed on duck nests opportunistically (Barding & Nelson, 2008). Raccoons moved relatively quickly through upland nesting habitats in other studies as well (Barding & Nelson, 2008; Greenwood, 1982; Rulison et al., 2012; Schoonover & Marshall, 1951). In contrast, skunks predominantly prey on insects and rodents (Crabtree & Wolfe, 1988; Dixon, 1925; Greenwood et al., 1999; Wade‐Smith & Verts, 1982). In areas outside of the core upland nesting area, with limited upland nesting habitat and a greater prevalence of seasonal wetlands, skunks and raccoons may use that landscape differently, but those movements and habitat associations would have less relevance for the vulnerability of upland‐nesting dabbling ducks to predation.

Both predator searching behavior and duck hen response to predators likely played a role in whether predators moving near a nest would discover nests and depredate eggs. Although individual raccoons encountered duck nests more often than skunks (combining both active and inactive nests), of those predators that encountered >1 nest, a higher percent of the nests encountered by skunks were depredated (52%) than those encountered by raccoons (22%). Of the active nests in our study, collared predators did not locate nests 96% of the time when they were within ≤25 m. We previously documented that hens often stay at their nest when approached by a predator, typically flushing only 29 s before a predator arrives at the nest (Croston, Ackerman, et al., 2018). Thus, encounters where the hen flushed off the nest and did not cover her eggs before she left the nest (4% of nest encounters; 77% of all departures) resulted in immediate nest failure (complete clutch depredation or abandonment) at 75% of nests when it was caused by a raccoon but only 40% of nests when it was caused by a skunk. Previously we found very high rates of partial clutch depredation (53% of depredated mallard nests; Ackerman et al., 2003) and that skunks tend to partially depredate duck nests whereas raccoons tend to completely destroy duck nests when they find them (Croston, Ackerman, et al., 2018). Remaining at the nest appears to be the preferred strategy for the hen to keep the nest hidden and is thought to provide protection for eggs (Forbes et al., 1994; Opermanis, 2004). We observed a few cases where the hen covered her eggs and left the nest early (1% of nest encounters; 20% of departures); those nests were discovered and depredated by a collared predator 17% of the time. Thus, covering the eggs and leaving the nest early when a predator is in the vicinity may be a less common but alternative strategy for avoiding predators at the nest. After the clutch has been completed, incubation recesses at night are relatively rare for dabbling ducks (14% of incubation recesses; Croston et al., 2021) and were presumed to occur primarily in response to a predator. However, 70% of nocturnal hen departures in a prior study in the same upland nesting area were cases where the hen covered her eggs and left the nest and it was unknown if a predator was in the vicinity of the nest at the time (Croston et al., 2021, 2020). Our new observations of hens covering the nest in response to a collared predator suggest that hens may proactively leave the nest at night 1% of the time when predators are within 25 m of the nest.

Although collared mammalian predators did not appear to gather in the core upland duck nesting habitat to exploit the seasonal pulse of eggs as a focal prey resource, individual predators showed high variability in their use of the core upland nesting area and individual specialization for egg predation may occur within these predator populations. Within the core upland nesting area, the distance to phragmites patches had the largest magnitude of effect on nest survival at the landscape level. Collared raccoons and skunks were observed using phragmites patches for denning and day resting sites; consequently, phragmites may be a habitat feature that attracts predators to the core upland area because of the physical structure it provides and removal of phragmites from the core upland nesting area may improve nest survival rates. Within the home ranges of collared predators, nests that were farther from canals, trees, and large shrubs were less likely to be encountered by raccoons and skunks, suggesting that management of those habitat features may also alter predator movements and nest encounter rates, and decrease the likelihood of nest discovery. Habitat management of the specific features within the upland nesting areas that are primarily used by mammalian predators could potentially reduce nest predation.

## AUTHOR CONTRIBUTIONS

**Sarah H. Peterson:** Conceptualization (lead); data curation (equal); formal analysis (lead); funding acquisition (equal); investigation (equal); methodology (lead); project administration (equal); resources (equal); software (equal); supervision (equal); validation (equal); visualization (lead); writing – original draft (lead); writing – review and editing (equal). **Joshua T. Ackerman:** Conceptualization (lead); data curation (equal); formal analysis (supporting); funding acquisition (lead); investigation (equal); methodology (equal); project administration (equal); resources (lead); software (equal); supervision (lead); validation (equal); visualization (equal); writing – original draft (supporting); writing – review and editing (equal). **Meghan P. Keating:** Conceptualization (supporting); data curation (supporting); investigation (supporting); methodology (supporting); project administration (supporting); writing – review and editing (supporting). **Carley R. Schacter:** Conceptualization (supporting); data curation (supporting); investigation (supporting); methodology (supporting); validation (supporting); visualization (supporting); writing – review and editing (supporting). **C. Alex Hartman:** Conceptualization (supporting); data curation (equal); formal analysis (supporting); funding acquisition (supporting); investigation (supporting); methodology (supporting); project administration (supporting); resources (supporting); software (supporting); supervision (supporting); validation (supporting); visualization (supporting); writing – review and editing (equal). **Michael L. Casazza:** Conceptualization (equal); funding acquisition (lead); investigation (supporting); methodology (supporting); project administration (equal); resources (lead); writing – review and editing (supporting). **Mark P. Herzog:** Conceptualization (supporting); data curation (equal); formal analysis (supporting); funding acquisition (supporting); investigation (supporting); methodology (supporting); project administration (supporting); resources (supporting); software (supporting); supervision (supporting); validation (supporting); visualization (supporting); writing – review and editing (equal).

## FUNDING INFORMATION

This research was funded by the California Department of Water Resources and the U.S. Geological Survey's Ecosystems Mission Area.

## CONFLICT OF INTEREST

The authors declare no conflicts of interest.

## Data Availability

The data in this article are in ScienceBase: https://doi.org/10.5066/P9W12VVX.

## References

[ece39329-bib-0001] Ackerman, J. T. (2002). Of mice and mallards: Positive indirect effects of coexisting prey on waterfowl nest success. Oikos, 3, 469–480.

[ece39329-bib-0002] Ackerman, J. T. , Blackmer, A. L. , & Eadie, J. M. (2004). Is predation on waterfowl nests density dependent? – Tests at three spatial scales. Oikos, 107, 128–140.

[ece39329-bib-0003] Ackerman, J. T. , Eadie, J. M. , Loughman, D. L. , Yarris, G. S. , & McLandress, M. R. (2003). The influence of partial clutch depredation on duckling production. Journal of Wildlife Management, 67, 576–587.

[ece39329-bib-0004] Ackerman, J. T. , Herzog, M. P. , Yarris, G. S. , Casazza, M. L. , Burns, E. W. , & Eadie, J. M. (2014). Waterfowl ecology and management. In P. B. Moyle , A. Manfree , & P. L. Fiedler (Eds.), Suisun marsh: Ecological history and possible futures (pp. 103–132). University of California Press.

[ece39329-bib-0005] Arnold, T. W. (2010). Uninformative parameters and model selection using Akaike's information criterion. Journal of Wildlife Management, 74, 1175–1178. 10.2193/2009-367

[ece39329-bib-0006] Barding, E. E. , & Nelson, T. A. (2008). Raccoons use habitat edges in northern Illinois. The American Midland Naturalist, 159, 394–402. 10.1674/0003-0031(2008)159[394:RUHEIN]2.0.CO;2

[ece39329-bib-0007] Bates, D. , Maechler, M. , Bolker, B. , Walker, S. , Christensen, R. H. B. , Singmann, H. , Dai, B. , Grothendieck, G. , & Green, P. (2015). Linear mixed‐effects models using “eigen” and S4 . R package v. 1.1‐9. R Foundation for Statistical Computing. Retrieved October 5, 2015, from http://CRAN.R‐project.org/package=lme4

[ece39329-bib-0008] Bivand, R. , & Rundel, C. (2020). Rgeos: Interface to geometry engine ‐ open source (‘GEOS’) . R package version 0.5‐5. https://CRAN.R‐project.org/package=rgeos

[ece39329-bib-0009] Bixler, A. , & Gittleman, J. L. (2000). Variation in home range and use of habitat in the striped skunk (*Mephitis mephitis*). Journal of Zoology, 251, 525–533. 10.1017/S0952836900008128

[ece39329-bib-0010] Burnham, K. P. , & Anderson, D. R. (2002). Model selection and multimodel inference: A practical information‐theoretic approach (2nd ed.). Springer‐Verlag.

[ece39329-bib-0011] Casazza, M. L. , Mcduie, F. , Lorenz, A. A. , Keiter, D. , Yee, J. , Overton, C. T. , Peterson, S. H. , Feldheim, C. L. , & Ackerman, J. T. (2020). Good prospects: High‐resolution telemetry data suggests novel brood site selection behaviour in waterfowl. Animal Behaviour, 164, 163–172. 10.1016/j.anbehav.2020.04.013

[ece39329-bib-0012] Chamberlain, M. J. , & Leopold, B. D. (2002). Spatio‐temporal relationships among adult raccoons (*Procyon lotor*) in Central Mississippi. The American Midland Naturalist, 148, 297–308. 10.1674/0003-0031(2002)148[0297:STRAAR]2.0.CO;2

[ece39329-bib-0013] Cooper, S. M. , Jhala, S. , Rollins, D. , & Feagin, R. A. (2015). Nocturnal movements and habitat selection of mesopredators encountering bobwhite nests. Wildlife Society Bulletin, 39, 138–146. 10.1002/wsb.499

[ece39329-bib-0014] Cowardin, L. M. , Gilmer, D. S. , & Shaiffer, C. W. (1985). Mallard recruitment in the agricultural environment of North Dakota. Wildlife Monographs, 92, 3–37.

[ece39329-bib-0015] Crabtree, R. L. , Broome, L. S. , & Wolfe, M. L. (1989). Effects of habitat characteristics on gadwall nest predation and nest‐site selection. Journal of Wildlife Management, 53, 129–137.

[ece39329-bib-0016] Crabtree, R. L. , & Wolfe, M. L. (1988). Effects of alternate prey on skunk predation of waterfowl nests. Wildlife Society Bulletin, 16, 163–169.

[ece39329-bib-0017] Croston, R. , Ackerman, J. T. , Herzog, M. P. , Kohl, J. D. , Hartman, C. A. , Peterson, S. H. , Overton, C. T. , Feldheim, C. L. , & Casazza, M. L. (2018). Duck nest depredation, predator behavior, and female response using video. Journal of Wildlife Management, 82, 1014–1025. 10.1002/jwmg.21444

[ece39329-bib-0018] Croston, R. , Hartman, C. A. , Herzog, M. P. , Casazza, M. L. , & Ackerman, J. T. (2018). A new approach to automated incubation recess detection using temperature loggers. The Condor, 120, 739–750. 10.1650/CONDOR-18-6.1

[ece39329-bib-0019] Croston, R. , Hartman, C. A. , Herzog, M. P. , Casazza, M. L. , Feldheim, C. L. , & Ackerman, J. T. (2020). Timing, frequency, and duration of incubation recesses in dabbling ducks. Ecology and Evolution, 10, 2513–2529. 10.1002/ece3.6078 32184998 PMC7069289

[ece39329-bib-0020] Croston, R. , Peterson, S. H. , Hartman, C. A. , Herzog, M. P. , Feldheim, C. L. , Casazza, M. L. , & Ackerman, J. T. (2021). Nocturnal incubation recess and flushing behavior by duck hens. Ecology and Evolution, 11, 7292–7301. 34188813 10.1002/ece3.7561 PMC8216913

[ece39329-bib-0021] DeGregorio, B. A. , Weatherhead, P. J. , & Sperry, J. H. (2014). Power lines, roads, and avian nest survival: Effects on predator identity and predation intensity. Ecology and Evolution, 4, 1589–1600. 10.1002/ece3.1049 24967077 PMC4063460

[ece39329-bib-0022] Dickie, M. , McNay, S. R. , Sutherland, G. D. , Cody, M. , & Avgar, T. (2020). Corridors or risk? Movement along, and use of, linear features varies predictably among large mammal predator and prey species. The Journal of Animal Ecology, 89, 623–634. 10.1111/1365-2656.13130 31648375 PMC7028095

[ece39329-bib-0023] Dixon, J. (1925). Food predilections of predatory and fur‐bearing mammals. Journal of Mammalogy, 6, 34–46.

[ece39329-bib-0024] Duong, T. , Wand, M. , Chacon, J. , & Gramaki, A. (2019). Kernel smoothing . R package v. 1.11.5. R Foundation for Statistical Computing. Retrieved September 10, 2019, from http://CRAN.R‐project.org/package=ks

[ece39329-bib-0025] Elfelt, M. , & Moen, R. (2014). Accuracy and location success of an ultralite GPS receiver (Technical Report No. NRRI/TR‐2014/32). Release 1.0. Natural Resource Research Institute.

[ece39329-bib-0026] Forbes, M. R. L. , Clark, R. G. , Weatherhead, P. J. , & Armstrong, T. (1994). Risk‐taking by female ducks: Intra‐ and interspecific tests of nest defense theory. Behavioral Ecology and Sociobiology, 34, 79–85.

[ece39329-bib-0027] Fritzell, E. K. (1978). Habitat use by prairie raccoons during the waterfowl breeding season. Journal of Wildlife Management, 42, 118–127.

[ece39329-bib-0028] Gorini, L. , Linnell, J. D. C. , May, R. , Panzacchi, M. , Boitani, L. , Odden, M. , & Nilsen, E. B. (2012). Habitat heterogeneity and mammalian predator – Prey interactions. Mammal Review, 42, 55–77.

[ece39329-bib-0029] Greenwood, R. J. (1982). Nocturnal activity and foraging of prairie raccoons (*Procyon lotor*) in North Dakota. The American Midland Naturalist, 107, 238–243.

[ece39329-bib-0030] Greenwood, R. J. , Sargeant, A. B. , Piehl, J. L. , Buhl, D. A. , & Hanson, B. A. (1999). Food and foraging of prairie striped skunks during the avian nesting season. Wildlife Society Bulletin, 27, 823–832.

[ece39329-bib-0031] Hannon, S. J. , & Cotterill, S. E. (1998). Nest predation in aspen woodlots in an agricultural area in Alberta: The enemy from within. The Auk, 115, 16–25.

[ece39329-bib-0032] Hoekman, S. , Mills, L. , Howerter, D. , Devries, J. , & Ball, I. (2002). Sensitivity analyses of the life cycle of midcontinent mallards. Journal of Wildlife Management, 66, 883–900.

[ece39329-bib-0033] Holling, C. S. (1959). The components of predation as revealed by a study of small mammal predation on the European pine sawfly. Canadian Entomologist, 91, 293–320.

[ece39329-bib-0034] Husby, M. , & Hoset, K. S. (2018). Seasonal variation in nest predation rates in boreal forests. Journal für Ornithologie, 159, 975–984. 10.1007/s10336-018-1563-y

[ece39329-bib-0035] Ibarzabal, J. , & Desrochers, A. (2004). A nest predator's view of a managed forest: Gray jay (*Perisoreus canadensis*) movement patterns in response to forest edges. The Auk, 121, 162–169.

[ece39329-bib-0036] James, A. R. C. , & Stuart‐Smith, A. K. (2000). Distribution of caribou and wolves in relation to linear corridors. Journal of Wildlife Management, 64, 154–159.

[ece39329-bib-0037] Johnson, D. H. D. H. (1979). Estimating nest success: The Mayfield method and an alternative. The Auk, 96, 651–661.

[ece39329-bib-0038] Klett, A. T. , Shaffer, T. L. , & Johnson, D. H. (1988). Duck nest success in the prairie pothole region. Journal of Wildlife Management, 52, 431–440. 10.2307/3801586

[ece39329-bib-0039] Korpimäki, E. , Norrdahl, K. , & Rinta‐Jaskari, T. (1991). Responses of stoats and least weasels to fluctuating food abundances: Is the low phase of the vole cycle due to mustelid predation? Oecologia, 88, 552–561. 10.1007/BF00317719 28312626

[ece39329-bib-0040] Lahti, D. C. (2001). The “edge effect on nest predation” hypothesis after twenty years. Biological Conservation, 99, 365–374. 10.1016/S0006-3207(00)00222-6

[ece39329-bib-0041] Larivière, S. , & Messier, F. (1998). Effect of density and nearest neighbours on simulated waterfowl nests: Can predators recognize high‐density nesting patches? Oikos, 83, 12–20. 10.2307/3546541

[ece39329-bib-0042] Larivière, S. , & Messier, F. (2000). Habitat selection and use of edges by striped skunks in the Canadian prairies. Canadian Journal of Zoology, 78, 366–372. 10.1139/z99-230

[ece39329-bib-0043] Larivière, S. , & Messier, F. (2016). Spatial organization of a prairie striped skunk population during the waterfowl nesting season. Journal of Wildlife Management, 62, 199–204.

[ece39329-bib-0044] Mauser, D. M. , Jarvis, R. L. , & Gilmer, D. S. (1994). Movements and habitat use of mallard broods in northeastern California. Journal of Wildlife Management, 58, 88–94. 10.2307/3809553

[ece39329-bib-0045] McKinnon, L. , Berteaux, D. , & Bêty, J. (2014). Predator‐mediated interactions between lemmings and shorebirds: A test of the alternative prey hypothesis. The Auk, 131, 619–628. 10.1642/AUK-13-154.1

[ece39329-bib-0046] McLandress, M. R. , Yarris, G. S. , Perkins, A. E. H. , Connelly, D. P. , & Raveling, D. G. (1996). Nesting biology of mallards in California. Journal of Wildlife Management, 60, 94–107.

[ece39329-bib-0047] Michelot, T. , Langrock, R. , & Patterson, T. A. (2016). moveHMM: An R package for the statistical modelling of animal movement data using hidden Markov models. Methods in Ecology and Evolution, 7, 1308–1315.

[ece39329-bib-0048] Nams, V. O. (1997). Density‐dependent predation by skunks using olfactory search images. Oecologia, 110, 440–448. 28307234 10.1007/s004420050179

[ece39329-bib-0049] Opermanis, O. (2004). Appearance and vulnerability of artificial duck nests to avian predators. Journal of Avian Biology, 35, 410–415.

[ece39329-bib-0050] Pedersen, E. J. , Miller, D. L. , Simpson, G. L. , & Ross, N. (2019). Hierarchical generalized additive models in ecology: An introduction with mgcv. PeerJ, 2019, e6876. 10.7717/peerj.6876 PMC6542350 31179172

[ece39329-bib-0051] Peterson, S. H. , & Ackerman, J. T. (2022). Predator movements and duck nests in relation to habitat features in Suisun marsh, CA (2016‐2019) . U.S. Geological Survey Data Release. 10.5066/P9W12VVX

[ece39329-bib-0052] Peterson, S. H. , Ackerman, J. T. , Hartman, C. A. , Casazza, M. L. , Feldheim, C. L. , & Herzog, M. P. (2021). Mercury exposure in mammalian mesopredators inhabiting a brackish marsh. Environmental Pollution, 273, 115808. 10.1016/j.envpol.2020.115808 33497946

[ece39329-bib-0053] Pieron, M. R. , & Rohwer, F. C. (2010). Effects of large‐scale predator reduction on nest success of upland nesting ducks. Journal of Wildlife Management, 74, 124–132. 10.2193/2009-056

[ece39329-bib-0054] Pitt, J. A. , Larivière, S. , & Messier, F. (2008). Social organization and group formation of raccoons at the edge of their distribution. Journal of Mammalogy, 89, 646–653.

[ece39329-bib-0055] Powell, L. A. (2007). Approximating variance of demographis parameters using the Delta method: A reference for avian biologists. The Condor, 109, 949–954.

[ece39329-bib-0056] Prange, S. , Gehrt, S. D. , & Hauver, S. (2011). Frequency and duration of contacts between free‐ranging raccoons: Uncovering a hidden social system. Journal of Mammalogy, 92, 1331–1342. 10.1644/10-MAMM-A-416.1

[ece39329-bib-0057] R Core Team . (2020). R: A language and environment for statistical computing. R Foundation for Statistical Computing. https://www.R‐project.org/

[ece39329-bib-0058] Raquel, A. J. , Ringelman, K. M. , Ackerman, J. T. , & Eadie, J. M. (2015). Habitat edges have weak effects on duck nest survival at local spatial scales. Ardea, 103, 155–162. 10.5253/arde.v103i2.a4

[ece39329-bib-0059] Ringelman, K. M. , Eadie, J. M. , & Ackerman, J. T. (2014). Adaptive nest clustering and density‐dependent nest survival in dabbling ducks. Oikos, 123, 239–247. 10.1111/j.1600-0706.2013.00851.x

[ece39329-bib-0060] Roos, S. (2002). Functional response, seasonal decline and landscape differences in nest predation risk. Oecologia, 133, 608–615. 10.1007/s00442-002-1056-8 28466159

[ece39329-bib-0061] Rulison, E. L. , Luiselli, L. , & Burke, R. L. (2012). Relative impacts of habitat and geography on raccoon diets. The American Midland Naturalist, 168, 231–246. 10.1674/0003-0031-168.2.231

[ece39329-bib-0062] Sacks, B. N. , Jaeger, M. M. , Neale, J. C. C. , & McCullough, D. R. (1999). Territoriality and breeding status of coyotes relative to sheep predation. Journal of Wildlife Management, 63, 593. 10.2307/3802648

[ece39329-bib-0063] San Francisco Estuary Institute and Aquatic Science Center (SFEI ASC) . (2017). Bay Area Aquatic Resource Inventory (BAARI) version 2.1 GIS data . Retrieved November 26, 2018, from http://www.sfei.org/data/baari‐version‐21‐gis‐data

[ece39329-bib-0064] Sargeant, A. B. , & Raveling, D. G. (1992). Mortality during the breeding season. In B. D. J. Batt , A. D. Afton , M. G. Anderson , C. D. Ankney , D. H. Johnson , J. A. Kadlec , & G. L. Krapu (Eds.), Ecology and management of breeding waterfowl (pp. 396–422). University of Minnesota Press.

[ece39329-bib-0065] Sargeant, A. B. , Sovada, M. A. , & Greenwood, R. J. (1998). Interpreting evidence of depredation of duck nests in the prairie pothole region. U.S. Department of the Interior, U.S. Geological Survey, Biological Resources Division.

[ece39329-bib-0066] Sargeant, A. B. , Sovada, M. A. , & Shaffer, T. L. (1995). Seasonal predator removal relative to hatch rate of duck nests in waterfowl production areas. Wildlife Society Bulletin, 23, 507–513.

[ece39329-bib-0067] Schacter, C. R. , Peterson, S. H. , Herzog, M. P. , Hartman, C. A. , Casazza, M. L. , & Ackerman, J. T. (2021). Wetland availability and salinity concentrations for breeding waterfowl in Suisun Marsh, California. San Francisco Estuary and Watershed Science, 19, 5–25.

[ece39329-bib-0068] Schmidt, K. A. , & Whelan, C. J. (1998). Predator‐mediated interactions between and within guilds of nesting songbirds: Experimental and observational evidence. The American Naturalist, 152, 393–402. 10.1086/286177 18811447

[ece39329-bib-0069] Schoonover, L. J. , & Marshall, W. H. (1951). Food habits of the raccoon (*Procyon lotor hirtus*) in north‐Central Minnesota. Journal of Mammalogy, 32, 422–428.

[ece39329-bib-0070] Seber, G. A. F. (1982). The estimation of animal abundance and related parameters (2nd ed.). Macmillan.

[ece39329-bib-0071] Shaffer, T. L. (2004). A unified approach to analyzing nest success. The Auk, 121, 526–540.

[ece39329-bib-0072] Shuford, D. , & Gardali, T. (2008). California bird species of special concern. Studies of western birds 1. Western Field Ornithologists and California Department of Fish and Game.

[ece39329-bib-0073] Singmann, H. , Bolker, B. , & Westfall, J. (2015). Afex: Analysis of factorial experiments . R package v. 0.14‐2. R Foundation for Statistical Computing. Retrieved July 28, 2015, from http://CRAN.R‐project.org/package=afex

[ece39329-bib-0074] Storm, G. L. (1972). Daytime retreats and movements of skunks on farmlands in Illinois. Journal of Wildlife Management, 36, 31–45.

[ece39329-bib-0075] Urban, D. (1970). Raccoon populations, movement patterns, and predation on a managed waterfowl marsh. Journal of Wildlife Management, 34, 372–382.

[ece39329-bib-0076] Vetter, D. , Rücker, G. , & Storch, I. (2013). A meta‐analysis of tropical forest edge effects on bird nest predation risk: Edge effects in avian nest predation. Biological Conservation, 159, 382–395. 10.1016/j.biocon.2012.12.023

[ece39329-bib-0077] Wade‐Smith, J. , & Verts, B. J. (1982). Mephitis mephitis . Mammalian Species, 173, 1–7.

[ece39329-bib-0078] Wehtje, M. , & Gompper, M. E. (2011). Effects of an experimentally clumped food resource on raccoon *Procyon lotor* home‐range use. Wildlife Biology, 17, 25–32. 10.2981/10-012

[ece39329-bib-0079] Weller, M. W. (1956). A simple field candler for waterfowl eggs. Journal of Wildlife Management, 20, 111–113. 10.2307/3797414

